# Formylation of Electron-Rich Aromatic Rings Mediated by Dichloromethyl Methyl Ether and TiCl_4_: Scope and Limitations

**DOI:** 10.3390/molecules20045409

**Published:** 2015-03-26

**Authors:** Iván Ramos-Tomillero, Marta Paradís-Bas, Ibério de Pinho Ribeiro Moreira, Josep María Bofill, Ernesto Nicolás, Fernando Albericio

**Affiliations:** 1 Institute for Research in Biomedicine (IRB Barcelona), Barcelona 08028, Spain; E-Mails: ivan.ramos@irbbarcelona.org (I.R.-T.); marta.paradis@irbbarcelona.org (M.P.-B.); 2 Deparment of Organic Chemistry, University of Barcelona, Barcelona 08028, Spain; E-Mail: jmbofill@ub.edu; 3 Department of Physical Chemistry, University of Barcelona, Barcelona 08028, Spain; E-Mail: i.moreira@ub.edu; 4 Institut de Química Teòrica i Computacional (IQTCUB), University of Barcelona, Barcelona 08028, Spain; 5 Institut de Biomedicina (IBUB), University of Barcelona, Barcelona 08028, Spain; 6 CIBER-BBN, Barcelona 08028, Spain; 7 School of Chemistry, University of KwaZulu-Natal, Durban 4000, South Africa; 8 School of Chemistry, Yachay Tech, Yachay City of Knowledge, Urcuqui 100119, Ecuador

**Keywords:** formylation, TiCl_4_, aromatic rings, aldehydes, dichloromethyl methyl ether

## Abstract

Here the aromatic formylation mediated by TiCl_4_ and dichloromethyl methyl ether previously described by our group has been explored for a wide range of aromatic rings, including phenols, methoxy- and methylbenzenes, as an excellent way to produce aromatic aldehydes. Here we determine that the regioselectivity of this process is highly promoted by the coordination between the atoms present in the aromatic moiety and those in the metal core.

## 1. Introduction

The high reactivity of aldehydes makes them a key functional group in organic chemistry. This group is widespread in Nature, and its use in the synthesis of natural products is noteworthy. Furthermore, as efficient electrophiles, aldehydes can undergo further transformations to be converted into an extensive range of functional groups, such as hydroxyls, carboxylic acids, double bonds, and alkanes, among others [1]. As a result of this feature, aldehydes are widely used as active pharmaceutical ingredients and also commonly found in food and cosmetics [2]. Thus the synthesis and manipulation of this kind of compound is a continuous focus of research. In this regard, there is increased interest in the development of mild and efficient methods for the introduction of the aldehyde moiety into organic structures [3,4,5,6,7,8,9,10,11]. Traditionally, these methods involve the oxidation of alcohols, the selective reduction of esters, the reductive ozonolysis of alkenes, and so on [12,13,14].

During recent years, our group has channeled much research effort into developing new strategies for solid-phase peptide synthesis (SPPS) [15,16,17,18,19]. Special attention has been devoted to the development of protecting groups and linkers, which are the cornerstones of SPPS. Most of these are based on the benzyl (Bzl) moiety. To make this moiety more acid-labile and therefore more user friendly, we and other groups have developed linkers [20] and protecting groups [19,21] based on electron-rich aromatic compounds. The relatively higher acid lability of these groups when compared with the naked benzyl group is due to the stability of the carbocation formed in the removal process [22]. In this regard, electron-rich aromatic aldehydes, which can be easily transformed into hydroxymethyl- or aminomethyl benzyl-type moieties, are key intermediates for the development of new protecting groups and/or linkers. There is currently a wide range of choice of approaches regarding the introduction of a formyl group into aromatic rings [3,6]; however, the organic chemist continues to face the challenge of formylation through C-C bond formation [23], which is the most convenient approach for the case of aromatic aldehydes. One of the most widely used procedures is the well-known Vilsmeier-Haack reaction [24], whereby, in the presence of *N,N*-dimethylformamide (DMF) and phosphorous oxychloride, the activated aromatic compound furnishes the corresponding aromatic aldehyde. Additionally, the procedures described by Duff [25] or Casiraghi-Skattebøl [26] are typically used for phenolic formylation.

**Scheme 1:**
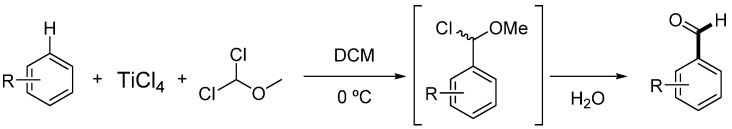
Titanium-mediated formylation reaction.

We have reported the successful *ortho*-formylation of electron-rich phenols mediated by dichloromethyl methyl ether and titanium (IV) tetrachloride [27], as well as a description of the reaction mechanisms in phenolic compounds [28]. This methodology was based on the outstanding procedure pioneered by Gross [29] and Cresp [30] that affords aromatic aldehydes (Scheme 1).

Herein we report an extensive study of this formylation procedure, where the dichloromethyl methyl ether (Cl_2_CHOMe) acts as a formylating agent for pre-activated aromatic rings in the presence of titanium tetrachloride.

## 2. Results and Discussion

**Scheme 2:**
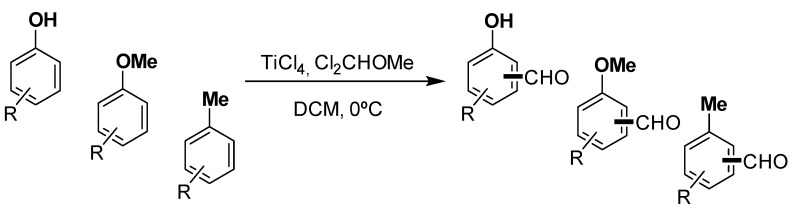
Formylated scaffolds examined in the present study.

Aiming to explore the range of applications of formylation by CHCl_2_OMe and TiCl_4_ in aromatic rings, we tested the reaction with three benzene-like activated rings. These substrates are structurally based on phenols, methoxy- and methylbenzenes (Scheme 2).

The general formylation procedure was carried out using the corresponding aromatic ring (1 eq.) mixed with TiCl_4_ (2.2 eq.) in dry dichloromethane (DCM) for 1 h under Ar at 0 °C. CHCl_2_OMe (1.1 eq.) was immediately added to the solution and stirred for 45 min, after which the reaction was quenched with a saturated aqueous solution of NH_4_Cl to furnish a mixture of regioisomers, giving satisfactory results in terms of regioselectivity, purity of the reaction crude products, and yields. This result contrasts with other common procedures that render less regioselectivity and more complex reaction crude products [24,25].

**Table 1:** Formylation of phenols, methoxy- and methylbenzenes.

Entry	Reactant	Formylation Conversion ^[a]^ %, [%]	Main Product		Regioisomeric Ratio ^[b]^ [*mp*: 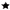 :▲]
			Aldehyde	Yield (%)	
**Phenols**					
1	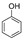	68 [22]	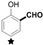	▬ ^[c]^	[2.8:1]
2	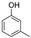	94 [6]	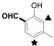	40 ^[d]^	[3:1.6:1]
3	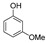	97	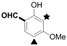	44 ^[d]^	[3.7:1.3:1]
4	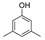	98 [2]	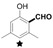	65	[5:1]
5	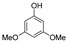	80 [11]	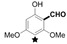	63	[30:1]
6	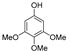	64 [25]	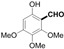	56	▬
**Methoxybenzenes**					
7	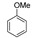	>99	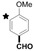	97 ^[d]^	[1.1:1]
8	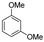	>99	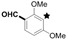	61	[3:1]
9	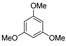	62 [38]	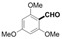	44	▬
10	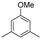	>99	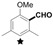	15	[3.5:1]	
**Methylbenzenes**					
11	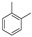	89 {4}	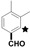	70 ^[d]^	[3.2:1]
12	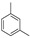	>99	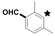	62 ^[d]^	[32:1]
13	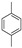	95% {3%}	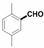	97	▬
14	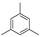	97	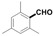	96	▬

Notes: ^[a]^ The % corresponds to the chromatographic peak area in the reaction crude determined by HPLC: total % of formylated products, [%] remaining starting material and {%} dimerization by-product. ^[b]^*mp*: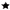:▲ indicates the ratio for the formylation of the main product and the other regioisomers. ^[c]^ Degradation during the purification. ^[d]^ The final products were isolated as mixture of regioisomers.

Table 1 provides a summary of the reactions. Some reactions furnish only one main product, as a result of the symmetry of the starting materials (entries 6, 9, 13 and 14). Meanwhile, non-symmetric starting reagents yielded distinct regioisomers.

### 2.1. Phenols

Regarding the formation of phenolic aldehydes (entries 1–6), the formylation takes place preferably in the *ortho* position with respect to the hydroxyl group (entries 1–5). This common behaviour is due to coordination between the metal atom and the oxygen of the OH, as previously studied by our group [27] and others [30]. After taking into account the role of the metal coordination, the steric hindrance caused by substituents also plays a significant role in formylation. Thus the formyl group was introduced in the less hindered position in each studied compound (entries 2, 3, 8, 10, 11 and 12).

### 2.2. Methoxybenzenes

In the case of the methoxy group (entries 7–10), Hamilton and co-workers demonstrated that TiCl_4_ are able to coordinate with several ethers [31], but it showed a weaker coordination effect in comparison with the hydroxyl one. Hence, the steric hindrance had a considerable effect on the formylated position. Thus for entry 7, the *para* substitution was favored, taking into account the presence of two *ortho* positions. The result in entry 7 reinforces the regioselectivity shown in entry 1. A special case of discussion is entry 10 *vs.* 7. In the first case, where the *ortho* and *para* positions were flanked by two substituents, the *ortho* one was favored. This observation indicates that both coordination and steric effects play a key role in the regioselectivity of formylation.

### 2.3. Methylbenzenes

**Scheme 3:**
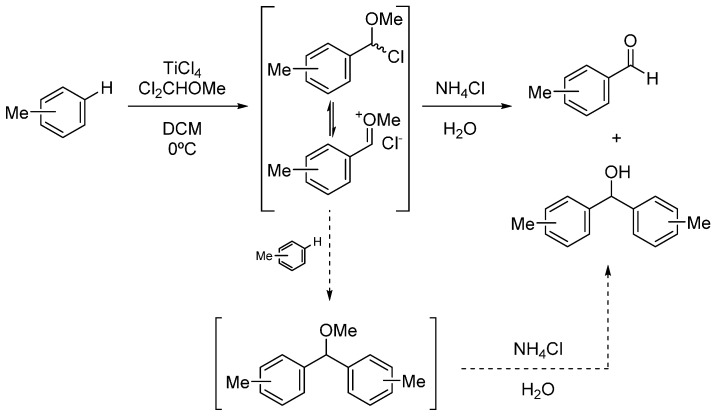
Proposed dimerization side-reaction mechanisms.

Concerning methylbenzene derivatives (entries 11–14), when formylation took place in the absence of oxygen atoms, coordination with the metal atom was not possible and therefore the less hindered isomer was favored. In this case, it is believed that the reaction mechanism occurs through the formation of an activated complex involving a π-interaction between the transition metal and the aromatic ring, as Calderazzo and co-workers proposed [32]. Indeed, another interesting point was the observed dimerization of two aromatic rings, affording diphenylmethanol compounds, as the proposed mechanism indicates (Scheme 3).

Accordingly, the chloride intermediate or the plausible oxocarbenium species [23] may undergo nucleophilic attack by an unreactive aromatic ring. This process provided the condensed diphenyl methoxy ether compound, which, with the acidolytic cleavage mediated by aqueous NH_4_Cl and the HCl obtained from the hydrolysis of TiCl_4_ (TiCl_4_ + 2 H_2_O → TiO_2_ + 4 HCl) [33,34], furnished the diphenylmethanol derivative in small quantities.

In general, most of the reactions tested showed high formylation conversions ranging from 64% to more than 99%. Accordingly, the titanium tetrachloride and Cl_2_CHOMe combination can be considered an effective formylating reagent.

## 3. Experimental Section

### 3.1. General Information

The commercially available products were used as received without further purification. Glass equipment was oven-dried, and DCM was dried using 4-Å molecular sieves under argon and protected from the light. All the reactions were carried out under argon. IR spectra were recorded on a Nicolet 510 FT-IR spectrometer equipped with an ATR Smart Orbit adaptor and are reported as frequency of absorption (cm^−1^). NMR spectra were recorded on a Varian Mercury-400 (^1^H/400 MHz and ^13^C/100 MHz). ^1^H data is reported as follows: chemical shift (δ ppm), [integration, multiplicity (s = singlet, d = doublet, t = triplet, q = quartet, m = multiplet), and coupling constant (*J* in Hz)]. Data for ^13^C-NMR are reported in terms of chemical shift. NMR spectra are referenced by tetramethylsilane (TMS). Melting points were measured with a Nikon Eclipse polarized microscope (MOP), which contains a Linkam THMS E600 thermal tray and a CI 93 temperature programmer. The HPLC reversed-phase column Xbridge C18 (75 × 4.6 mm, 3.5 μm) 4.6 × 3 × 150 mm, 5 µm was from Waters (Dublin, Ireland). Analytical HPLC (HPLC A and B) was carried out on using HPLC A: a Waters instrument comprising two solvent-delivery pumps (Waters 1525), an automatic injector (Waters 717 auto sampler), diode array wavelength detector (Waters 2487), and linear gradients of MeCN (+0.036% TFA) into H_2_O (+0.045% TFA) at 1 mL/min; or using HPLC B: a Shimadzu system comprising two solvent-delivery pumps (LC-20AD), an automatic injector (SIL-10ADvp), a variable wavelength detector (SPD-20A; 220 nm) and linear gradients of MeCN (+0.036% TFA) into H_2_O (+0.045% TFA) at 1 mL/min, which are specified in each case. The average in the chromatograms was determined by the area integration of the chromatographic peaks at λ = 220 nm. The thin-layer chromatography plates (TLC) used was purchased from Merck (TLC Silica gel 60 F_254_, silica-plated aluminium sheets). Column chromatography was performed on wet packed silica (Merck Silica gel 60, 0.2 mm). The automatic purification was performed by CombiFlash^®^ Rf Teledyne ISCO with a Waters detector 2487 Dual λ Absorbance using pre-packed Redisep Rf Gold C18 (20–40 μm, 100 Å) from Teledyne Technology Company.

### 3.2. General Formylation Procedure

**Figure 1:**
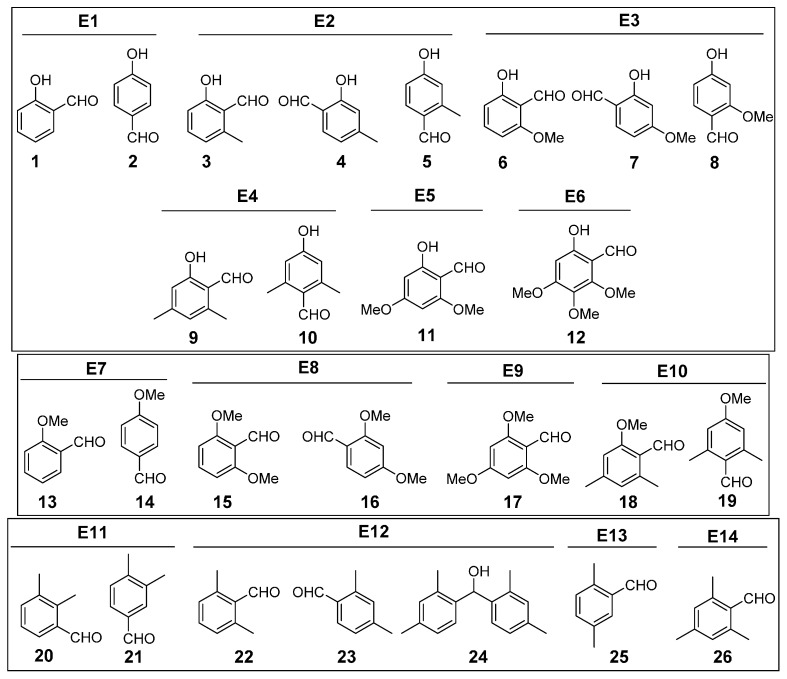
Obtained compounds.

The appropriate benzene derivative (3.2–10.6 mmol) was dissolved in dry DCM (10–20 mL), purged with Ar, and cooled with an ice bath to 0 °C. Next, TiCl_4_ (2.2 eq.) was added dropwise. The reaction mixture was stirred for 1 h. Afterwards, dichloromethyl methyl ether (1.1 eq.) was added, and the mixture was left to react for a further 45 min. As a reaction quencher, a saturated solution of NH_4_Cl (25 mL) was added. The mixture was then left for 2 h. The organic layer was separated and washed with 0.1 N HCl solution (3 × 50 mL) and brine (3 × 50 mL). The organic layer was dried over MgSO_4_ and filtered, and the solvent was evaporated under vacuum to furnish the desired aldehydes (Figure 1). The purified products were homogeneous by HPLC and were characterized and purified by using various physical techniques.

### 3.3. Phenols

#### 3.3.1. Entry 1

Reaction of phenol (1.0 g, 10.63 mmol), TiCl_4_ (2.6 mL, 26.71 mmol), dichloromethyl methyl ether (1.0 mL, 11.02 mmol) and DCM (20 mL) gave a purple oil. The products decomposed during SiO_2_ purification and only aldehyde **2** could be isolated (10 mg, 0.75%). HPLC detected 18%, 22% and 50% of 4-hydroxyaldehyde (**2**), the starting material, and 2-hydroxybenzaldehyde (**1**) respectively. *2-Hydroxybenzaldehyde* (**1**): HPLC (H_2_O/MeCN from 95:5 to 0:100 over 8 min): t_R_ = 4.9 min. *4-Hydroxybenzaldehyde* (**2**): Appearance: White solid. HPLC (H_2_O/MeCN from 95:5 to 0:100 over 8 min): t_R_ = 3.6 min. ^1^H-NMR (C_3_D_6_O) δ 9.86 (1H, s), 9.38 (1H, s), 7.83–7.77 (2H, m), 7.03–6.99 (2H, m) ppm. ^13^C-NMR (C_3_D_6_O) δ 191.0, 163.9, 132.8, 130.5, 116.7 ppm.

#### 3.3.2. Entry 2

Reaction of 3-methylphenol (336 μL, 3.21 mmol), TiCl_4_ (780 μL, 7.11 mmol), dichloromethyl methyl ether (320 μL, 3.54 mmol) and DCM (10 mL) afforded a pink solid. The mixture of the regioisomers was chromatographed on SiO_2_ (hexane/AcOEt, 9:1) to yield 4-hydroxy-2-methylbenzaldehyde (**5**) (69.8 mg, 16%) and a mixture of inseparable products, 2-hydroxy-6-methylbenzaldehyde (**3**) and 2-hydroxy-4-methylbenzaldehyde (**4**) in a 1:3.6 ratio, as determined by ^1^H-NMR (175.8 mg of the mixture was obtained, 40% yield of the formylation reaction). HPLC and ^1^H-NMR detected a small amount of the starting material (5.6%). *4-Hydroxy-2-methylbenzaldehyde* (**5**): Appearance: White solid. Mp = 106–108 °C. TLC: *Rf* = 0.1 (hexane/AcOEt, 9:1). HPLC (H_2_O/MeCN from 95:5 to 0:100 over 8 min): t_R_ = 4.1 min. ^1^H-NMR (C_3_D_6_O) δ 10.1 (1H, s), 9.21 (1H, s), 7.71 (1H, d, *J* = 8.4 Hz), 6.86–6.81 (1H, m), 6.78–6.74 (1H, m), 2.58 (4H, s) ppm. ^13^C-NMR (C_3_D_6_O) δ 191.3, 162.9, 144.0, 135.7, 128.3, 119.1, 114.1, 19.7 ppm. IR (ATR, Smart orbit): $\tilde{v}$ = 3118.7, 1657.5, 1556.0, 1307.4 ($\tilde{v}$_max_) cm^−1^.

#### 3.3.3. Entry 3

Reaction of 3-methoxyphenol (352 μL, 3.21 mmol), TiCl_4_ (780 μL, 7.11 mmol), dichloromethyl methyl ether (320 μL, 3.54 mmol) and DCM (10 mL) gave an orange solid. The mixture of regioisomers was chromatographed on SiO_2_ (hexane/AcOEt, 8:2) to yield 4-hydroxy-2-methoxy- benzaldehyde (**8**) (81.1 mg, 17%) and a mixture of inseparable products: 6-hydroxy-2-methoxy- benzaldehyde (**6**) and 2-hydroxy-4-methoxybenzaldehyde (**7**) in a 1:3.2 ratio determined by ^1^H-NMR (212.7 mg of the mixture was obtained, 44% yield of the formylation reaction). *4-Hydroxy-2-methoxybenzaldehyde* (**8**): Appearance: White solid. Mp = 155–157 °C. TLC: *R_f_* = 0.1 (hexane/AcOEt, 8:2). HPLC (H_2_O/MeCN from 95:5 to 0:100 over 8 min): *t*_R_ = 4.4 min. ^1^H-NMR (C_3_D_6_O) δ 10.2 (1H, s), 9.35 (1H, s), 7.64 (1H, d, *J =* 8.5 Hz), 6.57 (1H, d, *J =* 2.1 Hz), 6.53 (1H, ddd, *J =* 8.5, 2.1, 0.6 Hz), 3.91 (3H, s) ppm. ^13^C-NMR (C_3_D_6_O) δ 186.4, 164.7, 164.1, 129.7, 118.1, 108.2, 98.8, 55.2 ppm. IR (ATR, Smart orbit): $\tilde{v}$ = 3137.8, 1654.3, 1634.6, 1569.3 ($\tilde{v}$*_max_*) cm^−1^.

#### 3.3.4. Entry 4

Reaction of 3,5-dimethylphenol (395.8 mg, 3.24 mmol), TiCl_4_ (790 μL, 7.21 mmol), dichloromethyl methyl ether (330 μL, 3.65 mmol) and DCM (10 mL) gave an orange solid which was chromatographed on SiO_2_ (hexane/AcOEt, 9:1) to yield 2-hydroxy-4,6-dimethylbenzaldehyde (**9**) (318.2 mg, 65%) and 4-hydroxy-2,6-dimethylbenzaldehyde (**10**) (61.7 mg, 13%). The ratio of the products **9**:**10** was 5:1 and the global yield 78%. *2-Hydroxy-4,6-dimethylbenzaldehyde* (**9**): Appearance: Pale yellow solid. Mp = 48–50 °C. TLC: *R_f_* = 0.77 (hexane/AcOEt, 8:2). HPLC (H_2_O/MeCN from 95:5 to 0:100 over 8 min): *t*_R_ = 5.1 min. ^1^H-NMR (CDCl_3_) δ 11.9 (1H, m), 10.2 (1H, m), 6.62 (1H, s), 6.53 (1H, m), 2.55 (3H, s), 2.30 (3H, s) ppm. ^13^C-NMR (100 MHz, CDCl_3_) δ 194.7, 163.6, 149.4, 142.0, 123.3, 116.7, 116.3, 77.5, 77.2, 76.8, 22.3, 18.1 ppm. IR (ATR, Smart orbit): $\tilde{v}$ = 2879.8, 1640.8, 1625.6 ($\tilde{v}$*_max_*), 1567.2 cm^−1^. *4-Hydroxy-2,6-dimethylbenzaldehyde* (**10**): Appearance: White solid. Mp = 195–197 °C. TLC: *R_f_* = 0.23 (hexane/AcOEt, 8:2). HPLC (H_2_O/MeCN from 95:5 to 0:100 over 8 min): *t*_R_ = 6.9 min. ^1^H-NMR (C_3_D_6_O) δ 10.4 (1H, s), 6.60 (2H, s), 2.54–2.53 (6H, m). ^13^C-NMR (C_3_D_6_O) δ 206.1, 191.7, 145.3, 126.2, 117.2, 21.0 ppm. IR (ATR, Smart orbit): $\tilde{v}$ = 3138.5, 1649.9, 1602.3, 1555.7, 1313.9, 1151.3 ($\tilde{v}$ *_max_*) cm^−1^.

#### 3.3.5. Entry 5

Reaction of 3,5-dimethoxyphenol (502.8 mg, 3.26 mmol), TiCl_4_ (790 μL, 7.21 mmol), dichloromethyl methyl ether (330 μL, 3.65 mmol) and DCM (10 mL) afforded an orange solid, which was chromatographed on SiO_2_ (hexane/AcOEt, 9:1) to yield 6-hydroxy-2,4-dimethoxybenzaldehyde (**11**) (339.0 mg, 63%). *6-Hydroxy-2,4-dimethoxybenzaldehyde* (**11**): Appearance: Pale orange solid. Mp = 70–72 °C. TLC: *R_f_* = 0.28 (hexane/AcOEt, 8:2). HPLC B (H_2_O/MeCN from 95:5 to 0:100 over 11 min): *t*_R_ = 8.3 min. ^1^H-NMR (CDCl_3_) δ 12.5 (1H, s), 10.1 (1H, s), 6.01 (1H, d, *J =* 2.2 Hz), 5.91 (1H, d, *J =* 2.2 Hz), 3.85 (3H, s), 3.83 (3H, s) ppm. ^13^C-NMR (CDCl_3_) δ 191.8, 168.1, 166.4, 163.5, 106.0, 92.9, 90.6, 55.7, 55.7 ppm. IR (ATR, Smart orbit): $\tilde{v}$ = 1642.3, 1619.4, 600.7 ($\tilde{v}$*_max_*) cm^−1^.

#### 3.3.6. Entry 6

Reaction of 3,4,5-trimethoxyphenol (579.6 mg, 3.15 mmol), TiCl_4_ (760 μL, 6.93 mmol), dichloro-methyl methyl ether (320 μL, 3.54 mmol) and DCM (10 mL) provided a pale orange solid, which was chromatographed on SiO_2_ (DCM/hexane, 9:1) to yield 6-hydroxy-2,3,4-trimethoxybenzaldehyde (**12**) (372.0 mg, 56%). The HPLC analysis of the isolated mixture reveals 25% of starting material. *6-Hydroxy-2,3,4-trimethoxybenzaldehyde* (**12**)*:* Appearance: Pale yellow solid. Mp = 63–65 °C. TLC: *R_f_* = 0.52 (DCM/hexane, 9:1). HPLC B (H_2_O/MeCN from 95:5 to 0:100 over 11 min): *t*_R_ = 8.1 min. ^1^H-NMR (CDCl_3_) δ 12.1 (1H, s), 10.1 (1H, s), 6.19 (1H, s), 4.04 (3H, s), 3.90 (3H, s), 3.79 (3H, s) ppm. ^13^C-NMR (CDCl_3_) δ 192.8, 162.2, 161.3, 155.6, 134.0, 108.5, 95.4, 62.2, 61.4, 56.4 ppm. IR (ATR, Smart orbit): $\tilde{v}$ = 2945.4, 2850.4, 1638.5, 1623.2 ($\tilde{v}$*_max_*) cm^−1^.

### 3.4. Methoxybenzenes

#### 3.4.1. Entry 7

Reaction of anisole (352 μL, 3.21 mmol), TiCl_4_ (780 μL, 7.11 mmol), dichloromethyl methyl ether (320 μL, 3.54 mmol) and DCM (10 mL) were used to obtain a pale rosy oil that contained the two products. The mixture were not possible to isolate by SiO_2_ and ratio of 1:1.1 was determined by ^1^H-NMR for 2-methoxybenzaldehyde (**13**) and 4-methoxybenzaldehyde (**14**) respectively (424.4 mg, 97% global yield). *2-Methoxybenzaldehyde and 4-methoxybenzaldehyde* (**13** and **14**)*:* TLC: *R_f_* = 0.43–0.37 (DCM/hexane, 1:1). HPLC (H_2_O/MeCN from 95:5 to 0:100 over 8 min): *t*_R_ (13) = 6.0 min, *t*_R_ (14) = 6.3 min. ^1^H-NMR and ^13^C-NMR see SI.

#### 3.4.2. Entry 8

Reaction of 1,3-dimethoxybenzene (420 μL, 3.21 mmol), TiCl_4_ (780 μL, 7.11 mmol), dichloro-methyl methyl ether (320 μL, 3.54 mmol) and DCM (10 mL) gave a grey solid, which was purified on SiO_2_ (DCM) to yield 2,6-dimethoxybenzaldehyde (**15**) (97.4 mg, 18%) and 2,4-dimethoxybenzaldehyde (**16**) (323.9 mg, 61%). The ratio of regioisomers **16:15** was 3:1 and the global yield 79%. *2,6-Dimethoxybenzaldehyde* (**15**): Appearance: Pale yellow solid. Mp = 94–97 °C. TLC: *R_f_* = 0.21 (DCM). HPLC (H_2_O/MeCN from 95:5 to 0:100 over 8 min): *t*_R_ = 4.9 min. ^1^H-NMR (CDCl_3_) δ 10.5 (1H, s), 7.45 (1H, t, *J* = 8.5 Hz), 6.58 (2H, d, *J =* 8.5 Hz), 3.90 (6H, s) ppm. ^13^C-NMR (CDCl_3_) δ 189.6, 162.4, 136.0, 114.5, 104.0, 56.2 ppm. IR (ATR, Smart orbit): $\tilde{v}$ = 2844.9, 2796.7, 1671.7, 1589.9, 1576.0, 1109.6 ($\tilde{v}$) cm^−1^. *2,4-Dimethoxybenzaldehyde* (**16**): Appearance: White solid. Mp = 68–70 °C. TLC: *R_f_* = 0.51 (DCM). HPLC (H_2_O/MeCN from 95:5 to 0:100 over 8 min): *t*_R_ = 5.7 min. ^1^H-NMR (CDCl_3_) δ 10.3 (1H, d, *J =* 0.7 Hz), 7.80 (1H, d, *J =* 8.7 Hz), 6.54 (1H, ddd, *J =* 8.7, 2.2, 0.7 Hz), 6.44 (1H, d, *J =* 2.2 Hz), 3.90 (3H, s), 3.87 (3H, s) ppm. ^13^C-NMR (CDCl_3_) δ 188.5, 166.3, 163.8, 130.9, 119.2, 105.9, 98.1, 55.8, 55.7 ppm. IR (ATR, Smart orbit): $\tilde{v}$ = 2855.6, 2779.3, 1663.6, 1596.3, 1578.4, 826.5 ($\tilde{v}$*_max_*) cm^−1^.

#### 3.4.3. Entry 9

Reaction of 1,3,5-trimethoxybenzene (533.5 mg, 3.17 mmol), TiCl_4_ (760 μL, 6.93 mmol), dichloromethyl methyl ether (320 μL, 3.54 mmol) and DCM(10 mL), afforded a purple solid, which was purified on SiO_2_ (DCM/hexane, 9:1) to yield 2,4,6-trimethoxybenzaldehyde (**17**) (275.0 mg, 44%). *2,4,6-Trimethoxybenzaldehyde* (**17**): Appearance: Orange solid. Mp = 118–121 °C. TLC: *R_f_* = 0.37 (DCM/hexane, 9:1). HPLC B (H_2_O/MeCN from 95:5 to 0:100 over 11 min): *t*_R_ = 7.0 min. ^1^H-NMR (CDCl_3_) δ 10.4 (1H, s), 6.07 (2H, s), 3.88 (6H, s), 3.87 (3H, s) ppm. ^13^C-NMR (CDCl_3_) δ 187.9, 166.3, 164.2, 109.0, 90.4, 56.1, 55.6 ppm. IR (ATR, Smart orbit): $\tilde{v}$ = 2843.2, 2796.0, 1661.3, 1596.1, 1574.3, 806.6 ($\tilde{v}$*_max_*) cm^−1^.

#### 3.4.4. Entry 10

Reaction of 3,5-dimethylanisole (453 μL, 3.21 mmol), TiCl_4_ (780 μL, 7.11 mmol), dichloromethyl methyl ether (320 μL, 3.54 mmol) and DCM (10 mL), gave a violet oily solid, which was purified by reverse-phase liquid chromatography (Semipreparative HPLC: Xbridge Prep BEH 130 C18, 5 μm OBD^TM^ 19 × 100 mm column, gradient H_2_O/MeCN from 55:45 to 50:50 over 30 min) to yield a 3.5:1 ratio of 2-methoxy-4,6-dimethylbenzaldehyde (**18**) (77.4 mg, 14.7%) and 4-methoxy-2,6-dimethylbenzaldehyde (**19**) (22.1 mg, 4.2%) respectively. *2-Methoxy-4,6-dimethylbenzaldehyde* (**18**): Appearance: Yellow solid. Mp = 48–50 °C. TLC: *R_f_* = 0.50 (DCM). HPLC (H_2_O/MeCN from 95:5 to 0:100 over 8 min): *t*_R_ = 5.6 min. ^1^H-NMR (CDCl_3_) δ 10.6 (1H, s), 6.63 (1H, s), 6.62 (1H, s), 3.88 (3H, s), 2.54 (3H, s), 2.35 (3H, s) ppm. ^13^C-NMR (CDCl_3_) δ 191.9, 163.5, 145.8, 142.2, 125.2, 121.1, 109.9, 55.9, 22.3, 21.6 ppm. IR (ATR, Smart orbit): $\tilde{v}$ = 2965.3, 2926.5, 1671.3, 1598.5, 1319.1, 1146.5 cm^−1^. *4-Methoxy-2,6-dimethylbenzaldehyde* (**19**): Appearance: White solid. Mp = 49–50 °C. TLC: *R_f_* = 0.24 (DCM). HPLC (H_2_O/MeCN from 95:5 to 0:100 over 8 min): *t*_R_ = 4.7 min. ^1^H-NMR (CDCl_3_) 10.5 (1H, s), 6.59 (2H, s), 3.84 (3H, s), 2.61 (6H, s) ppm. ^13^C-NMR (CDCl_3_) 191.8, 162.9, 144.6, 126.2, 115.0, 55.4, 21.3 ppm. IR (ATR, Smart orbit):$\tilde{v}$ = 2960.2, 2921.5, 1678.8, 1610.3, 1465.1, 1299.4, 1205.1, 1097.2, 836.8 ($\tilde{v}$*_max_*) cm^−1^.

### 3.5. Methylbenzenes

#### 3.5.1. Entry 11

Reaction of *o-*xylene (386 μL, 3.20 mmol), TiCl_4_ (780 μL, 7.11 mmol), dichloromethyl methyl ether (320 μL, 3.54 mmol) and DCM (10 mL), afforded a green oil, which was purified on SiO_2_ (hexane/AcOEt, 8:2). During the purification, partial decomposition was observed. Both regioisomers were isolated as a 3.2:1 mixture of 3,4-dimethylbenzaldehyde (**21**) and 2,3-dimethylbenzaldehyde (**20**) respectively, determined by the ^1^H-NMR of the isolated mixture (see SI). TLC: *R_f_* = 0.48–0.53 (hexane/AcOEt, 8:2). HPLC (H_2_O/MeCN from 95:5 to 0:100 over 8 min): *t*_R_ = 6.7 min.

#### 3.5.2. Entry 12

Reaction of *m-*xylene (394 μL, 3.21 mmol), TiCl_4_ (780 μL (7.11 mmol), dichloromethyl methyl ether (320 μL, 3.54 mmol) and DCM (10 mL), afforded a yellow oil, which was purified on SiO_2_ (treated with 1% of NEt_3_) (hexane/AcOEt, 19:1) to yield a mixture of 2,6-dimethylbenzaldehyde (**22**) and 2,4-dimethylbenzaldehyde (**23**) (267.0 mg, 62%) in a 1:32 ratio, as determined by ^1^H-NMR of the isolated mixture. Moreover, bis(2,4-dimethylphenyl)methanol (**24**) was isolated as a dimerization by-product (38.0 mg, 9.9%). *Mixture of* **22** *and* **23**: TLC: *R_f_* = 0.25–0.27 (hexane/AcOEt, 19:1). HPLC (H_2_O/MeCN from 95:5 to 0:100 over 8 min): *t*_R_ = 6.8 min. *bis(2,4-Dimethylphenyl)methanol* (**24**): Appearance: White solid. TLC: *R_f_* = 0.12 (hexane/EtOAc, 19:1). HPLC (H_2_O/MeCN from 95:5 to 0:100 over 8 min): *t*_R_ = 8.3 min. ^1^H-NMR (CDCl_3_) δ 7.17 (2H, m), 6.99 (4H, m), 6.07 (1H, s), 2.32 (6H, s), 2.25 (6H, s) ppm. ^13^C-NMR (CDCl_3_) δ 138.2, 137.2, 135.7, 131.4, 126.8, 126.6, 70.1, 21.1, 19.1 ppm.

#### 3.5.3. Entry 13

Reaction of *p-*xylene (394 μL, 3.21 mmol), TiCl_4_ (780 μL, 7.11 mmol), dichloromethyl methyl ether (320 μL, 3.54 mmol) and DCM (10 mL), gave a rosy oil which was not purified (95% purity). The dimerization byproduct (3.4% of the peak area) was observed in the chromatographic traces. *2,5-Dimethylbenzaldehyde* (**25**): Appearance: Rosy oil. TLC: *R_f_* = 0.25–0.27 (hexane/EtOAc, 19:1). HPLC (H_2_O/MeCN from 95:5 to 0:100 over 8 min): *t*_R_ = 6.9 min. ^1^H-NMR (400 MHz, CDCl_3_) δ 10.23 (1H, s), 7.59 (1H, d, *J* = 1.2 Hz), 7.28 (1H, dd, *J* = 7.8, 1.5 Hz), 7.14 (1H, d, *J* = 7.8 Hz), 2.61 (3H, s), 2.37 (3H, s) ppm. ^13^C-NMR (100 MHz, CDCl_3_) δ 192.4, 144.6, 140.7, 132.6, 132.5, 132.0, 127.1, 21.7, 19.6 ppm. IR (ATR, Smart orbit): $\tilde{v}$ = 2923.8, 2722.1, 1690.5 ($\tilde{v}$*_max_*), 1611.3, 1504.9, 1240.7, 1155.2 cm^−1^.

#### 3.5.4. Entry 14

Reaction of mesytilene (1000 μL, 7.19 mmol), TiCl_4_ (1.5 mL, 13.68 mmol), dichloromethyl methyl ether (720 μL, 7.96 mmol) and DCM (25 mL), gave a colorless oil (1.02 g, 96%), which was not purified (97% purity). *2,4,6-Trimethylbenzaldehyde* (**26**): Appearance: Colorless oil. TLC: *R_f_* = 0.54 (DCM/hexane, 1:1). HPLC (H_2_O/MeCN from 95:5 to 0:100 over 8 min): *t*_R_ = 6.0 min. ^1^H-NMR (CDCl_3_) δ 10.5 (1H, s), 6.87 (2H, s), 2.55 (6H, s), 2.29 (3H, s) ppm. ^13^C-NMR (CDCl_3_) δ 192.87, 143.78, 141.43, 130.51, 129.95, 21.43, 20.46 ppm. IR (NaCl): $\tilde{v}$ =2921.4, 2865.3, 2783.1, 1687.1 ($\tilde{v}$*_max_*), 1608.4 cm^−1^.

## 4. Conclusions

In short, the formylation studies presented here demonstrate the potential of aromatic formylation using TiCl_4_ and dichloromethyl methyl ether as a straightforward and versatile reaction that affords a wide range of functionalized aldehydes. Of note, only for phenol derivatives did the oxygen-metal interaction contribute significantly to determining *o*-formylation. We consider that this reaction will allow the development of a new set of protecting groups and linkers for further application in SPPS.

## Supplementary Materials

Supplementary materials can be accessed at: http://www.mdpi.com/1420-3049/20/04/5409/s1.
