# Supplementary material for: Formylation of Electron-Rich Aromatic Rings Mediated by Dichloromethyl Methyl Ether and TiCl4: Scope and Limitations
Source: Molecules. 2015 Mar 26;20(4):5409–22. doi: 10.3390/molecules20045409 (PMC6272369; doi:10.3390/molecules20045409)
Supplement: Supplementary file 1 [file molecules-20-05409-s001.pdf]

Supplementary Materials

Table of Contents

1. **Nuclear magnetic resonance (NMR)**
2. **High performance liquid chromatography (HPLC): Reaction crudes**
3. **Resume table of the results for the formylation reaction.**

1. NMR SPECTRA

➢ **Entry 1: Reaction with phenol**

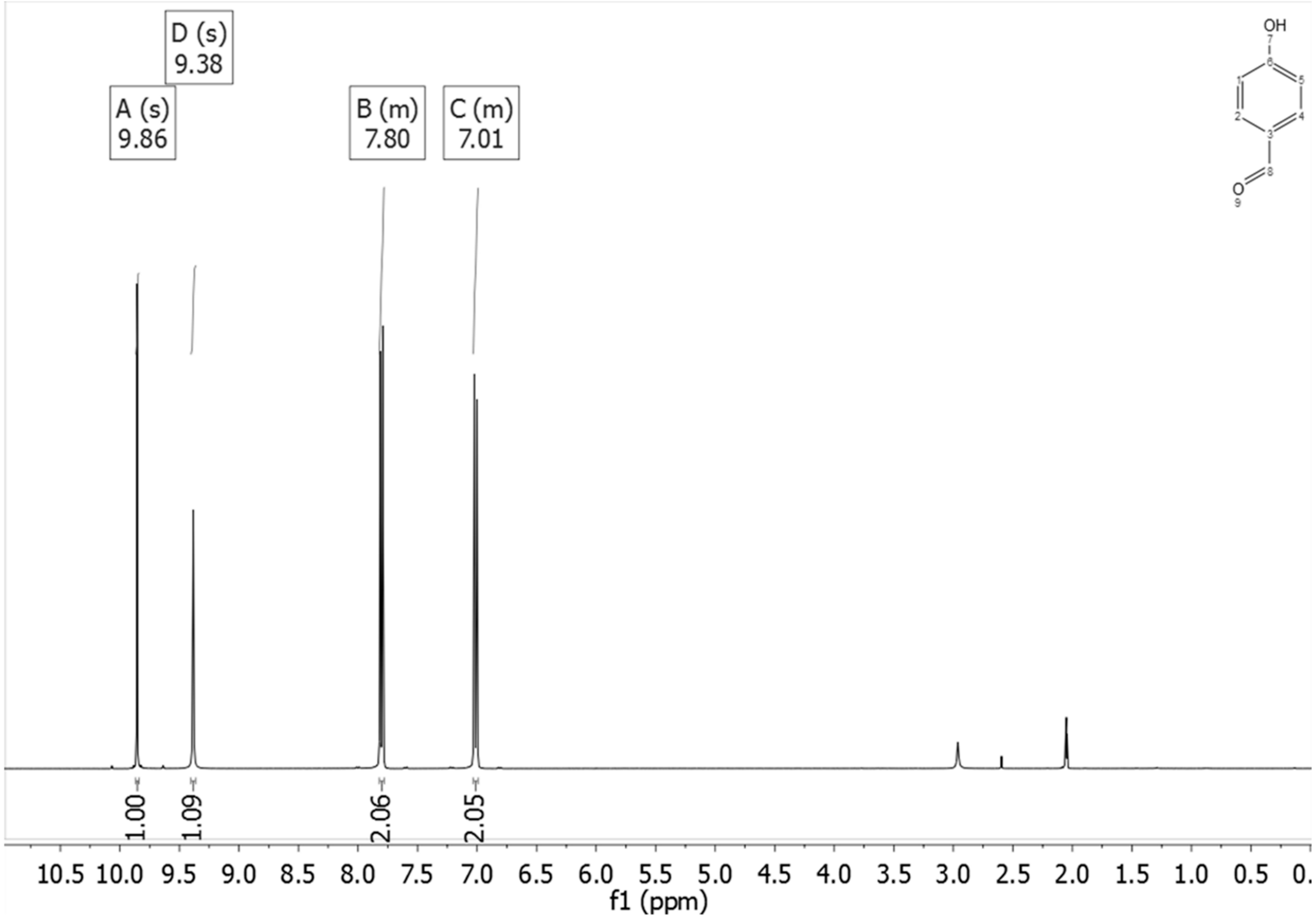

Figure S1. ^{1}H-NMR (400 MHz, C_3D_6O): **2**.

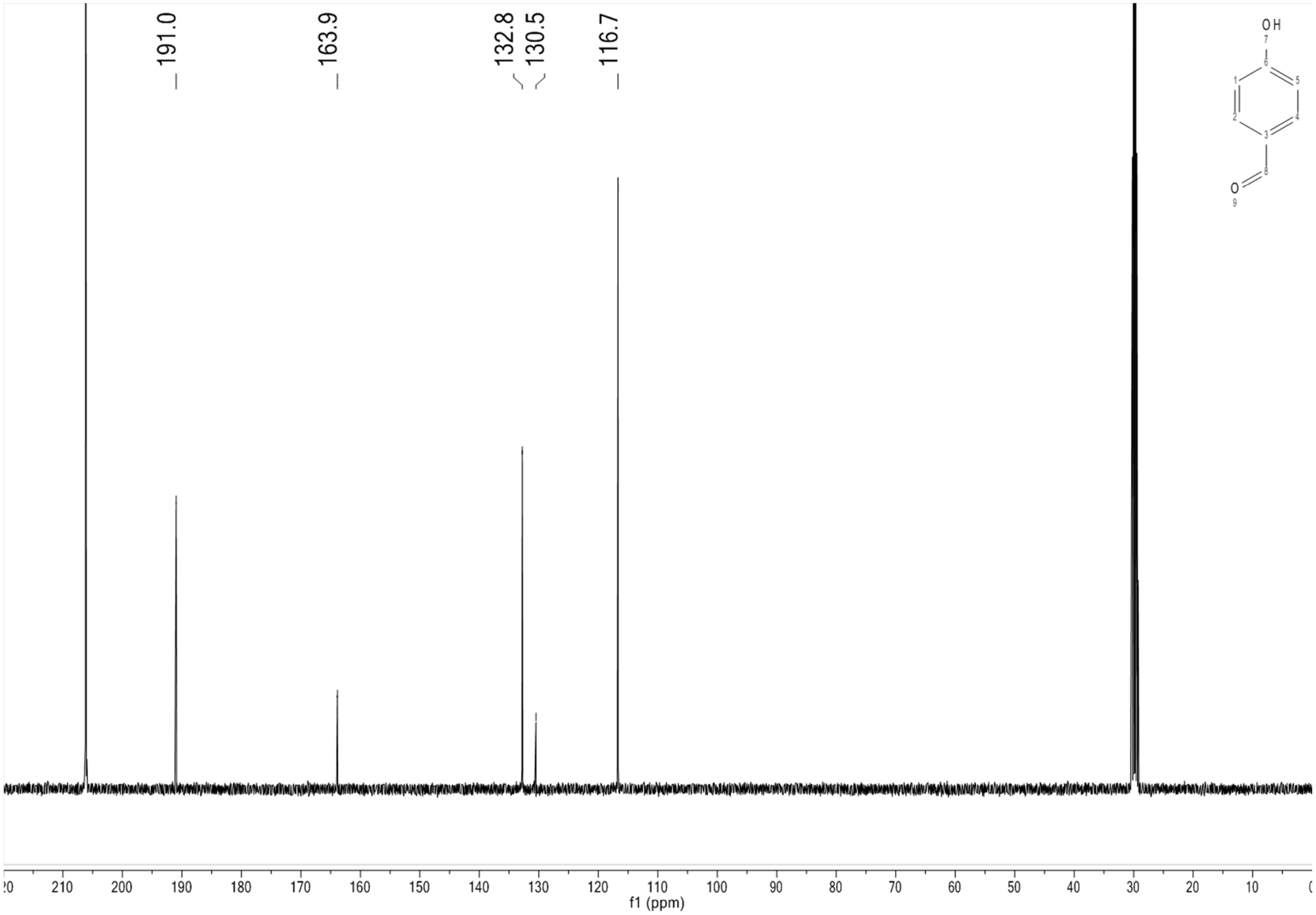

Figure S2. ^{13}C-NMR (100 MHz, C_3D_6O): **2**.

➢ **Entry 2: Reaction with 3-methylphenol**

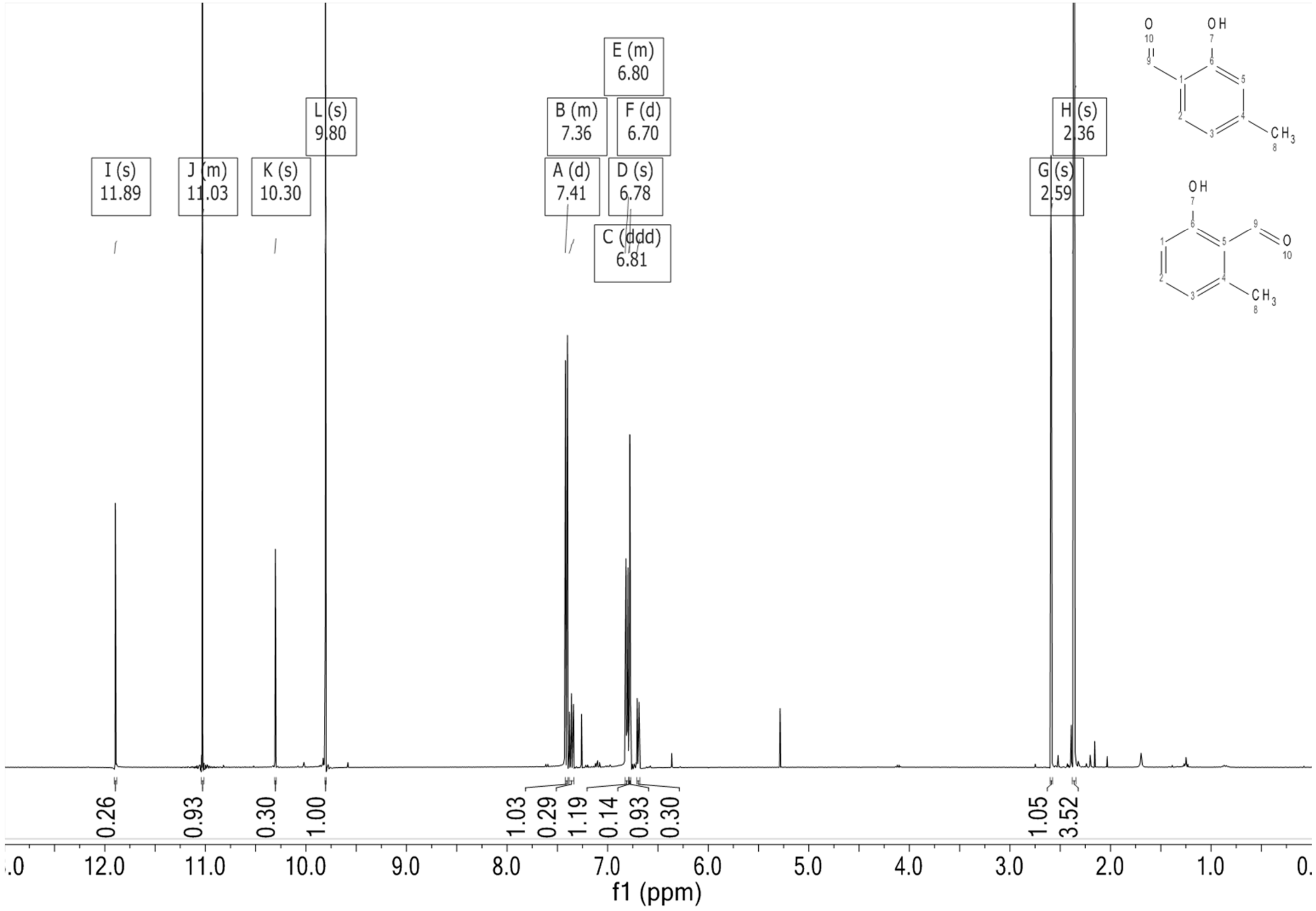

Figure S3. ^{1}H-NMR (400 MHz, C_3D_3O): **3** and **4**.

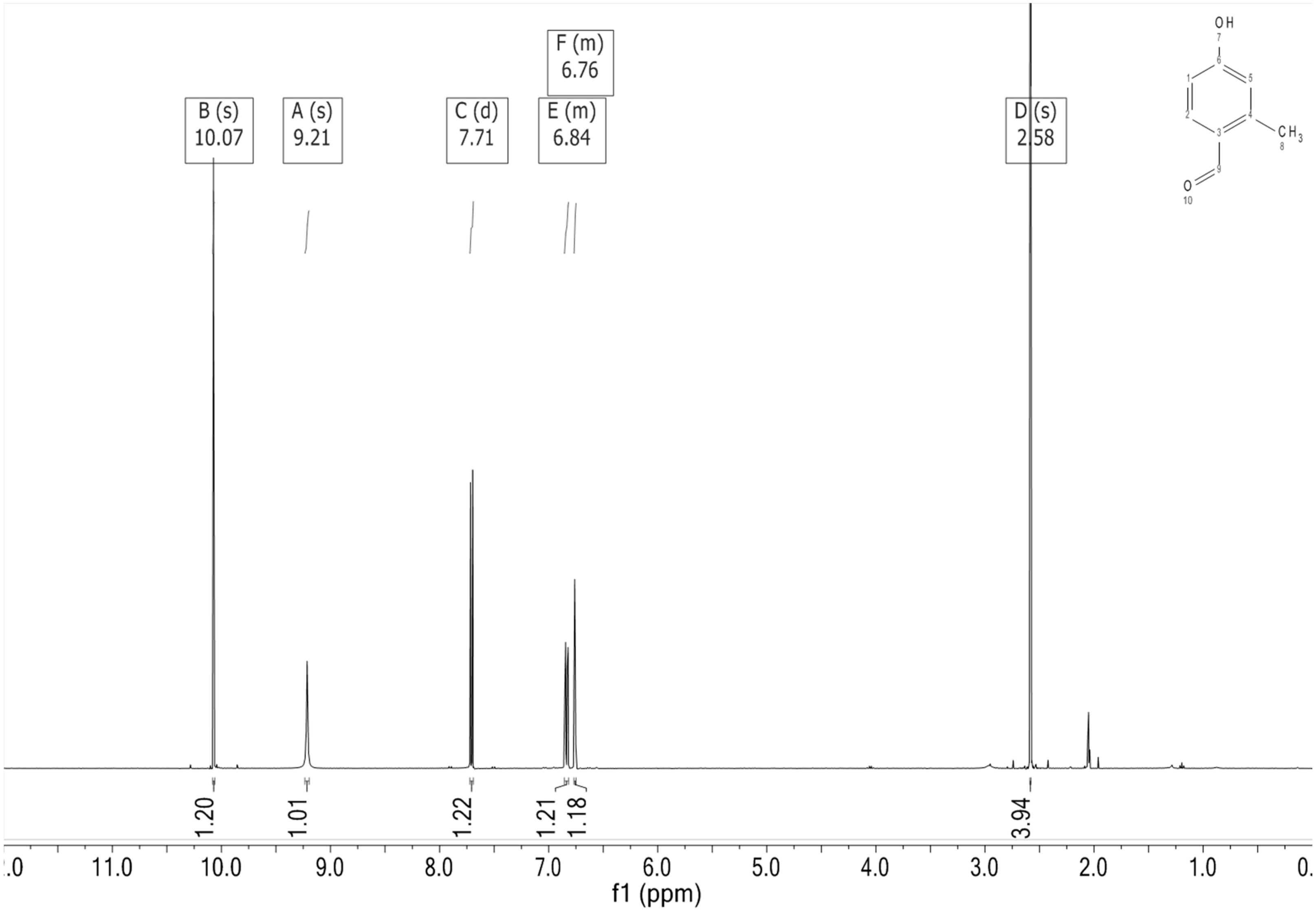

Figure S4. ^{1}H-NMR (400 MHz, C_3D_3O): **5**.

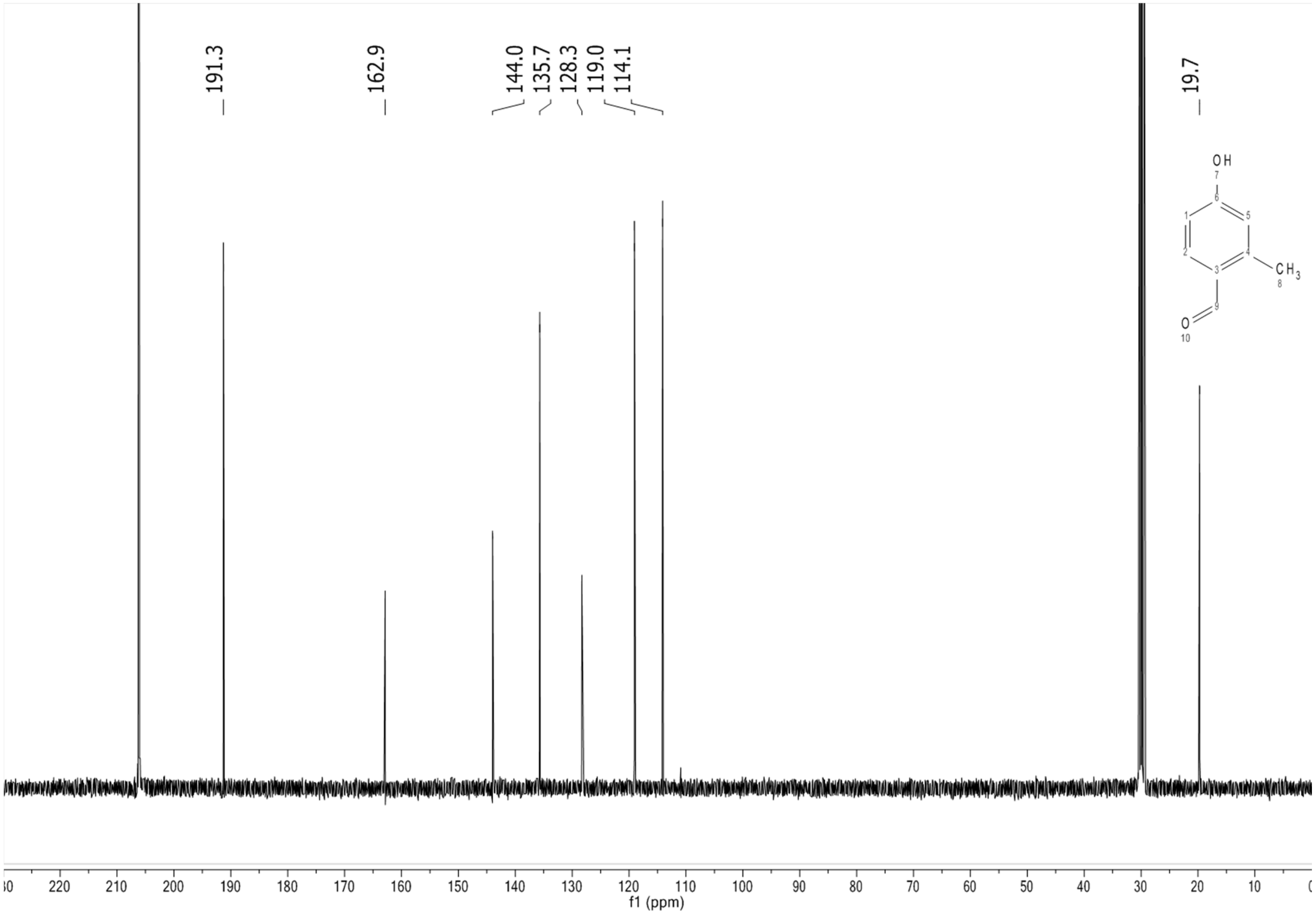

Figure S5. ^{13}C-NMR (100 MHz, C_3D_6O): **5**.

- **Entry 3: Reaction with 3-methoxyphenol**

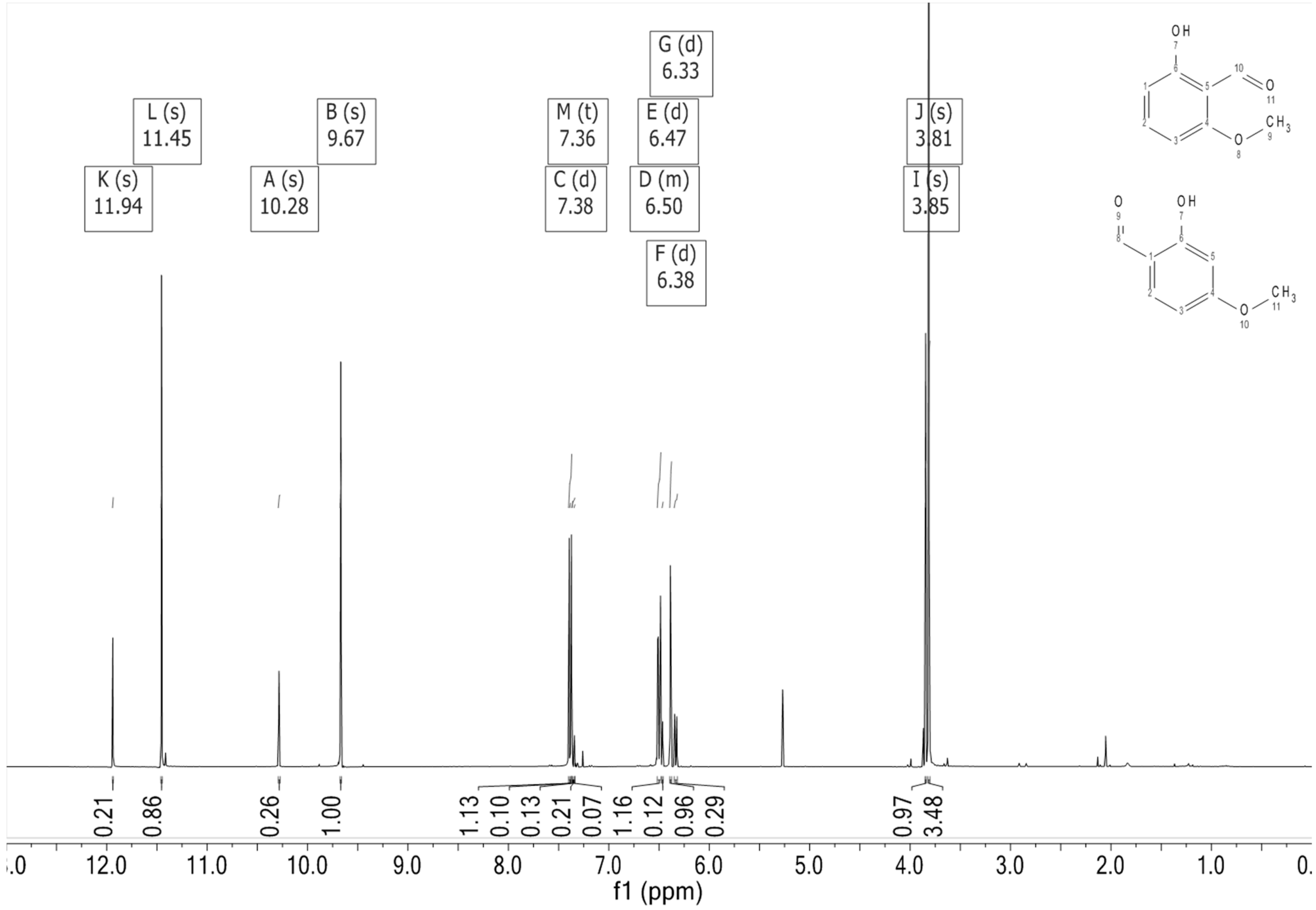

Figure S6. ^{1}H-NMR (400 MHz, C_3D_3O): **6** and **7**.

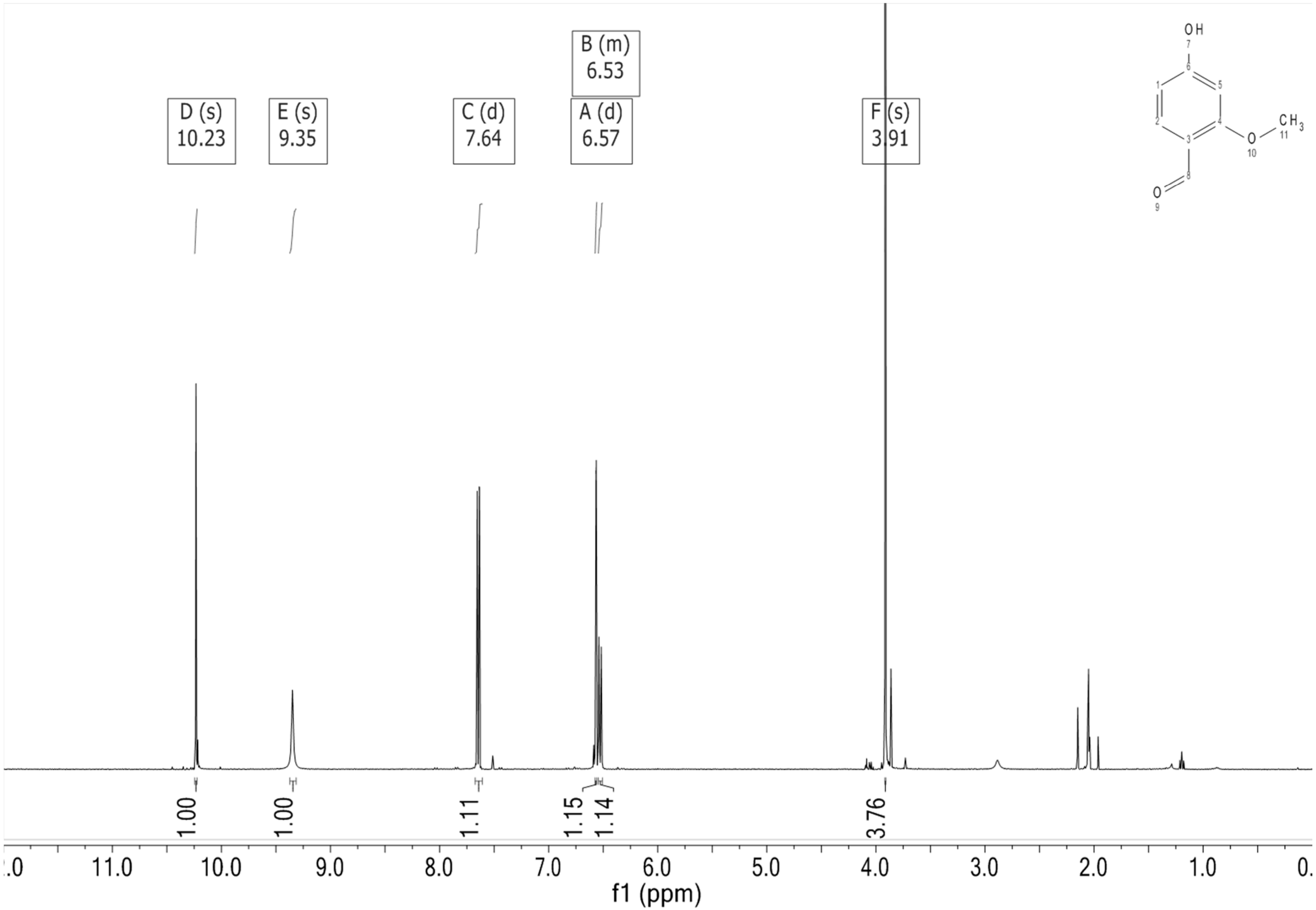

Figure S7. ^{1}H-NMR (400 MHz, C_3D_3O): **8**.

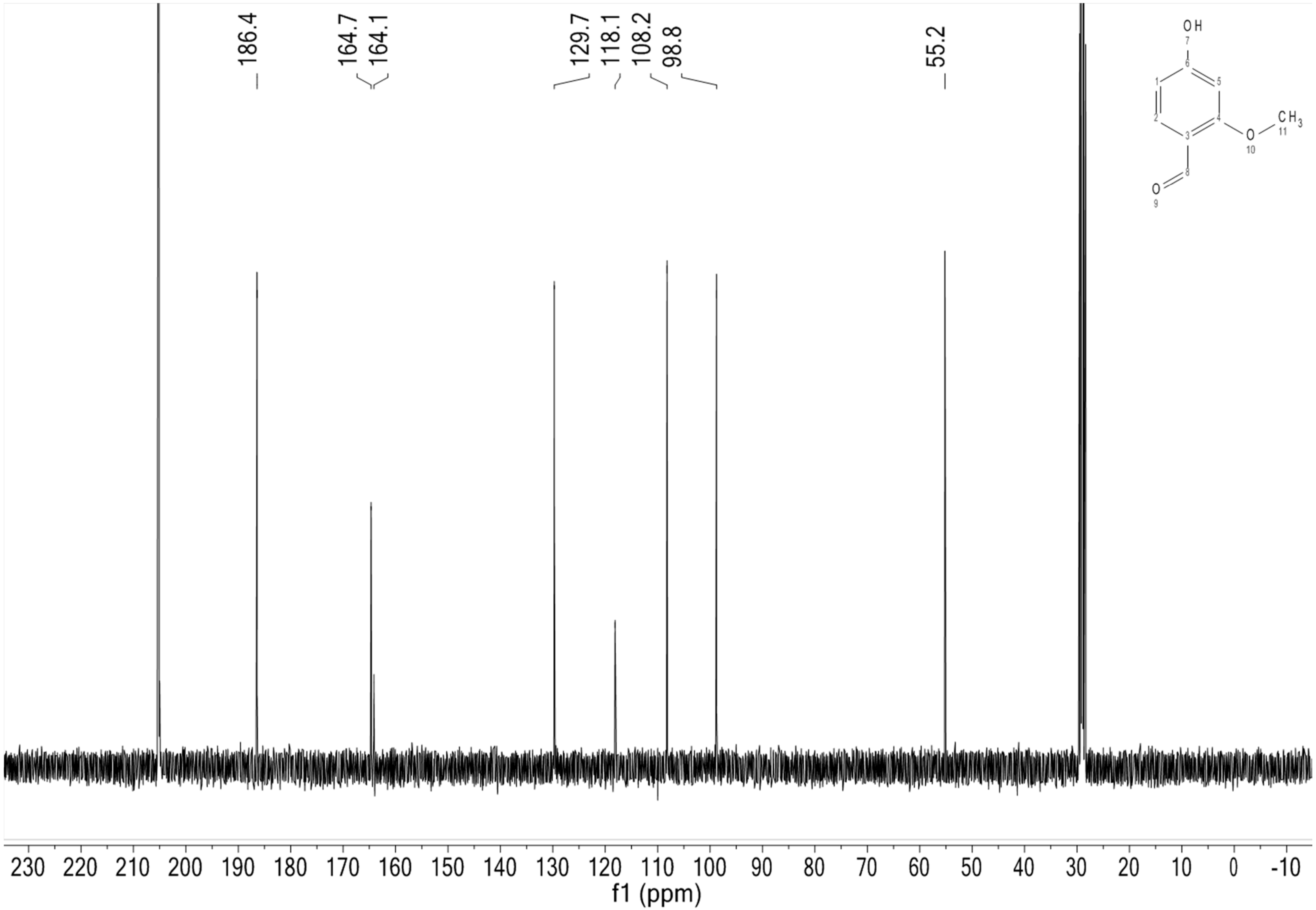

Figure S8. ^{13}C-NMR (100 MHz, C_3D_3O): **8**.

- **Entry 4: Reaction with 3,5-dimethylphenol**

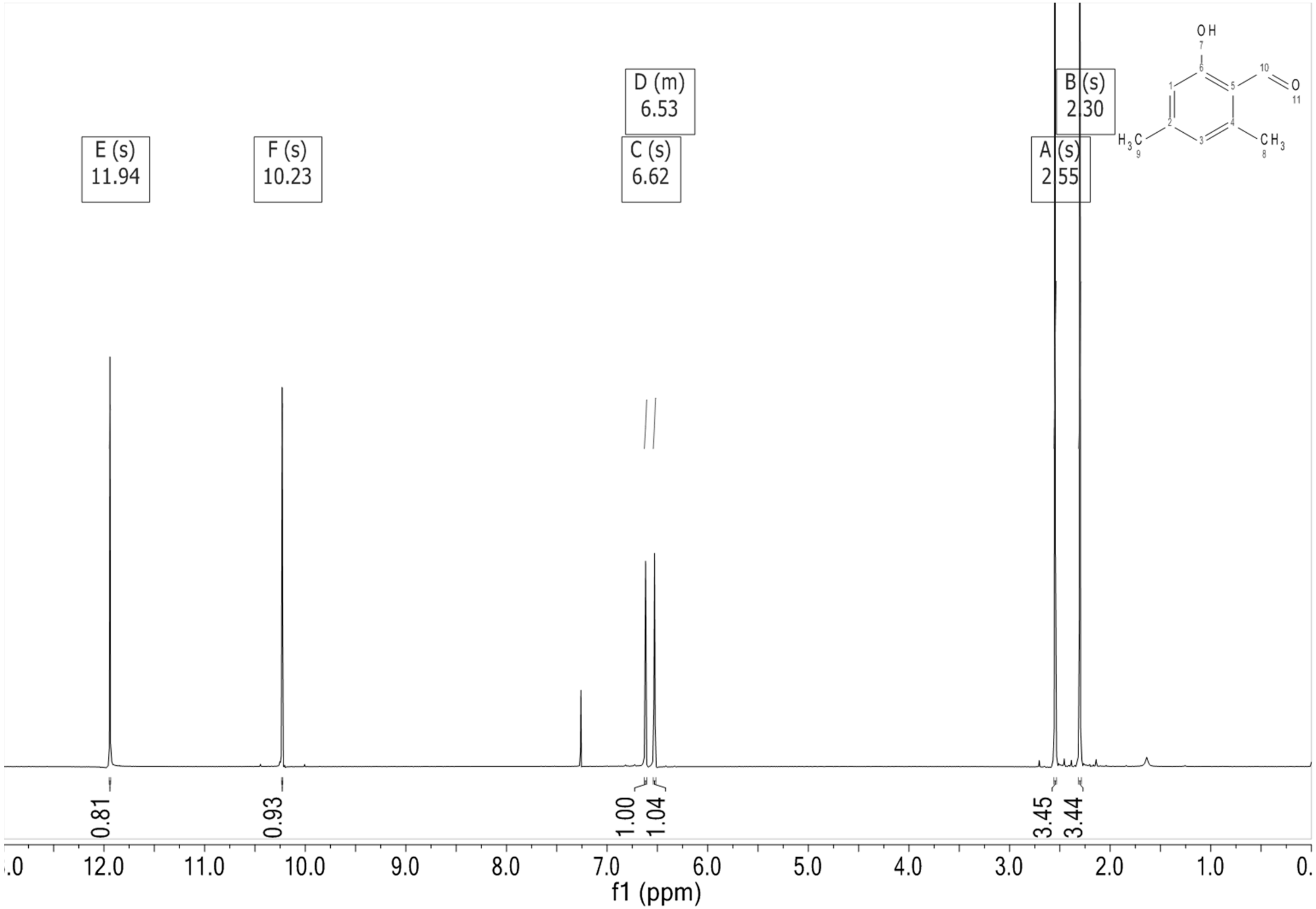

Figure S9. ^{1}H-NMR (400 MHz, $CDCl_3$): **9**.

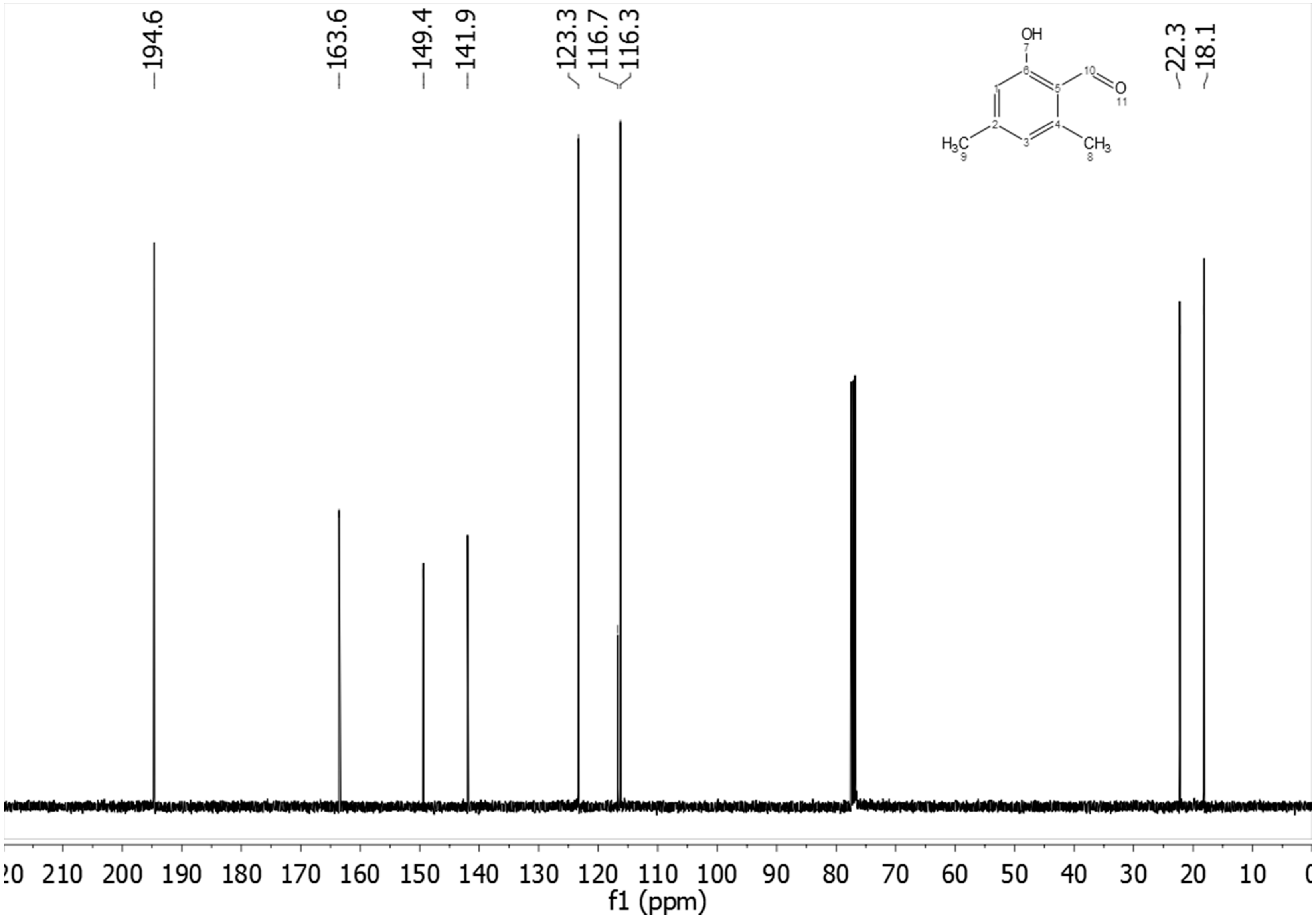

Figure S10. ^{13}C-NMR (100 MHz, $CDCl_3$): **9**.

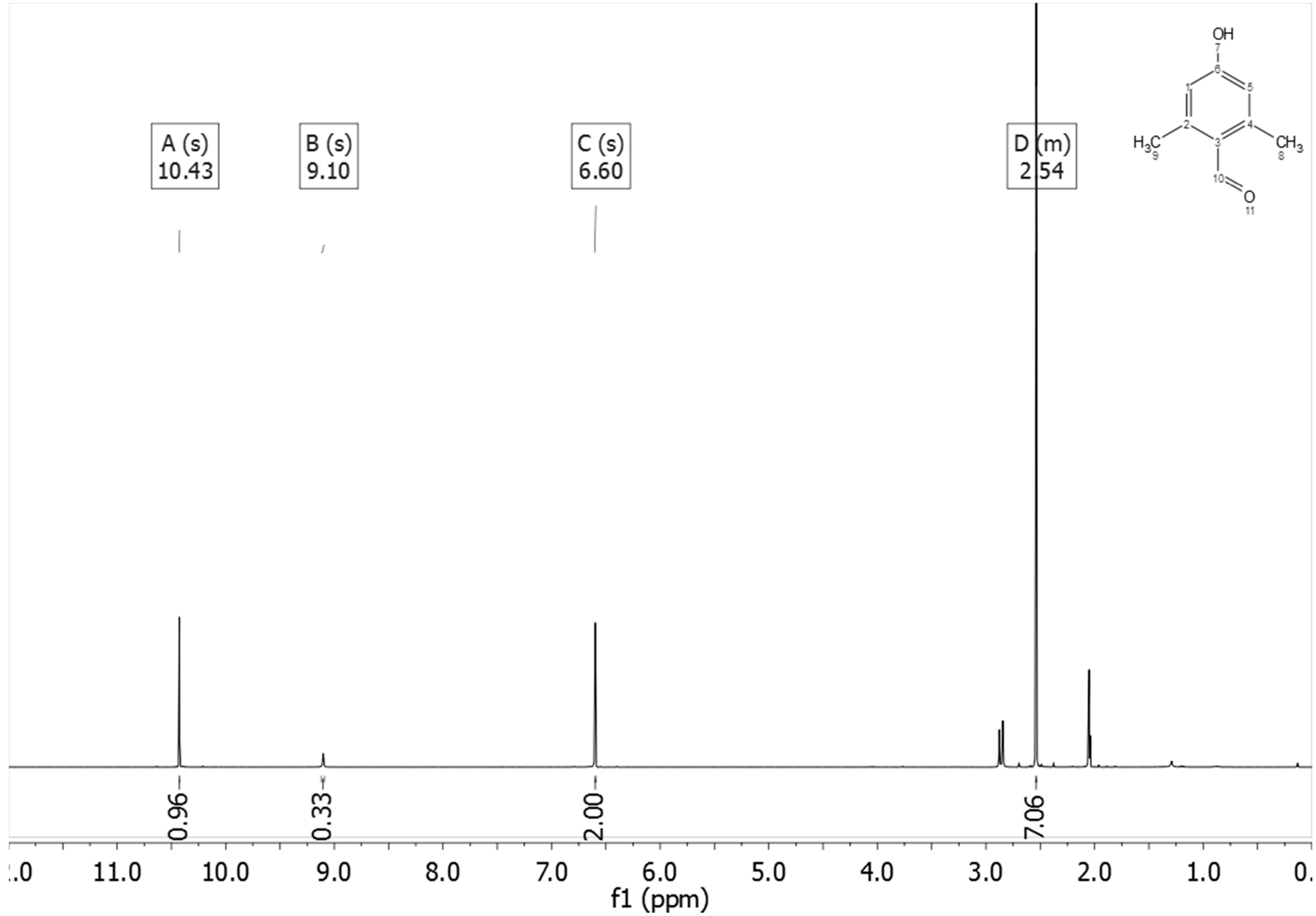

Figure S11. ^{1}H-NMR (400 MHz, C_3D_6O): **10**.

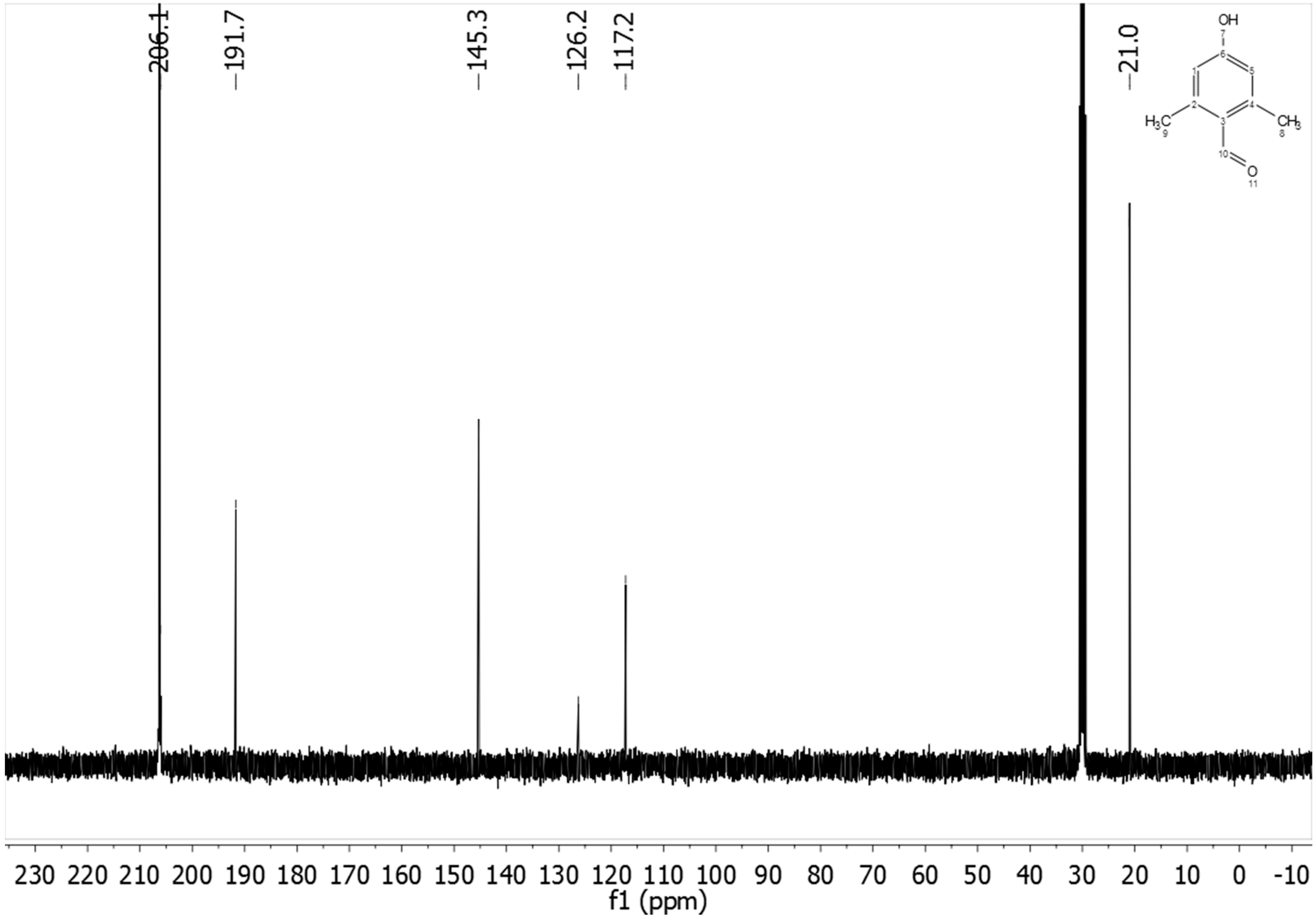

Figure S12. ^{13}C-NMR (100 MHz, C_3D_6O): **10**.

- **Entry 5: Reaction with 3,5-dimethoxyphenol:**

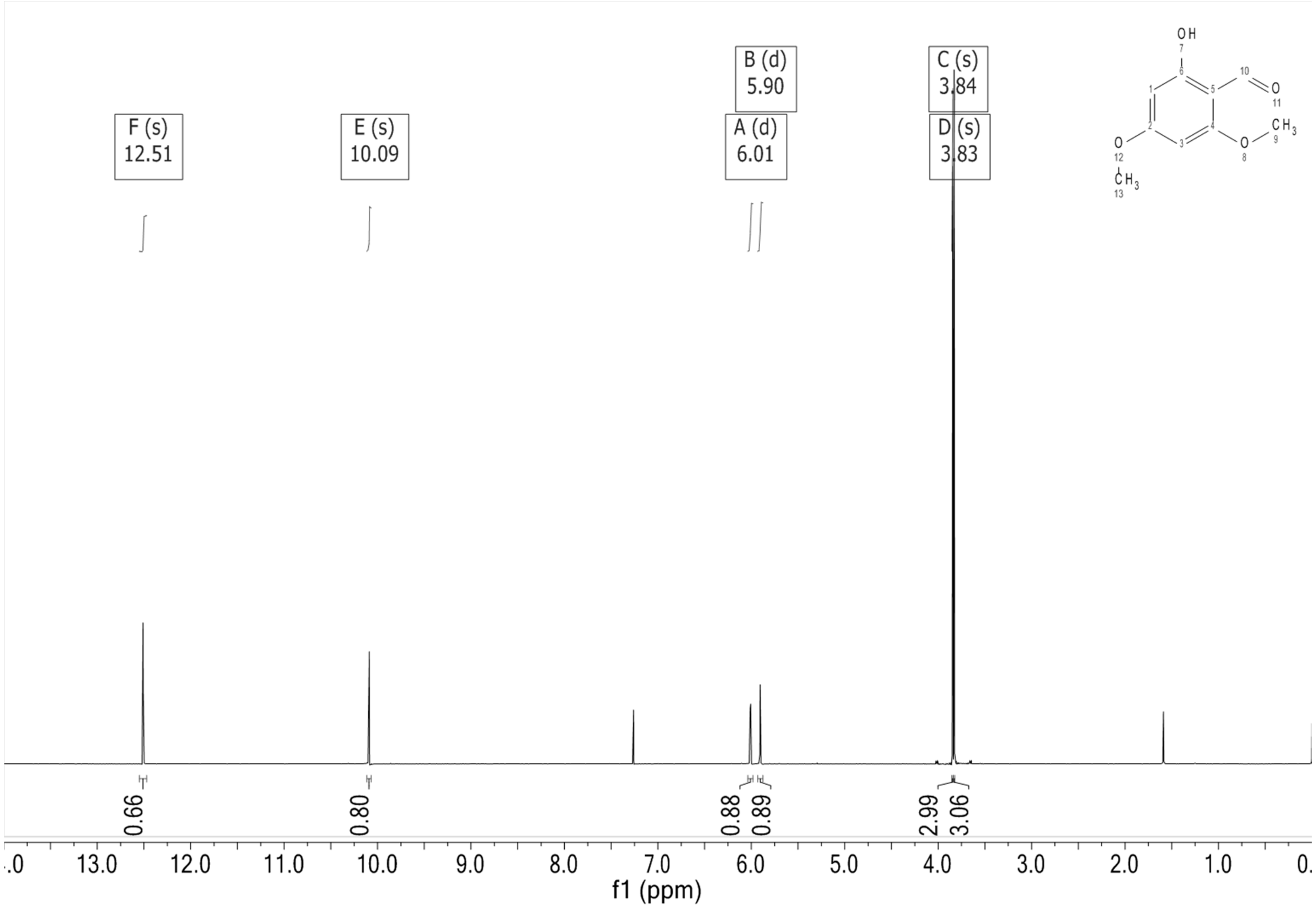

Figure S13. ^{1}H-NMR (400 MHz, $CDCl_3$): **11**.

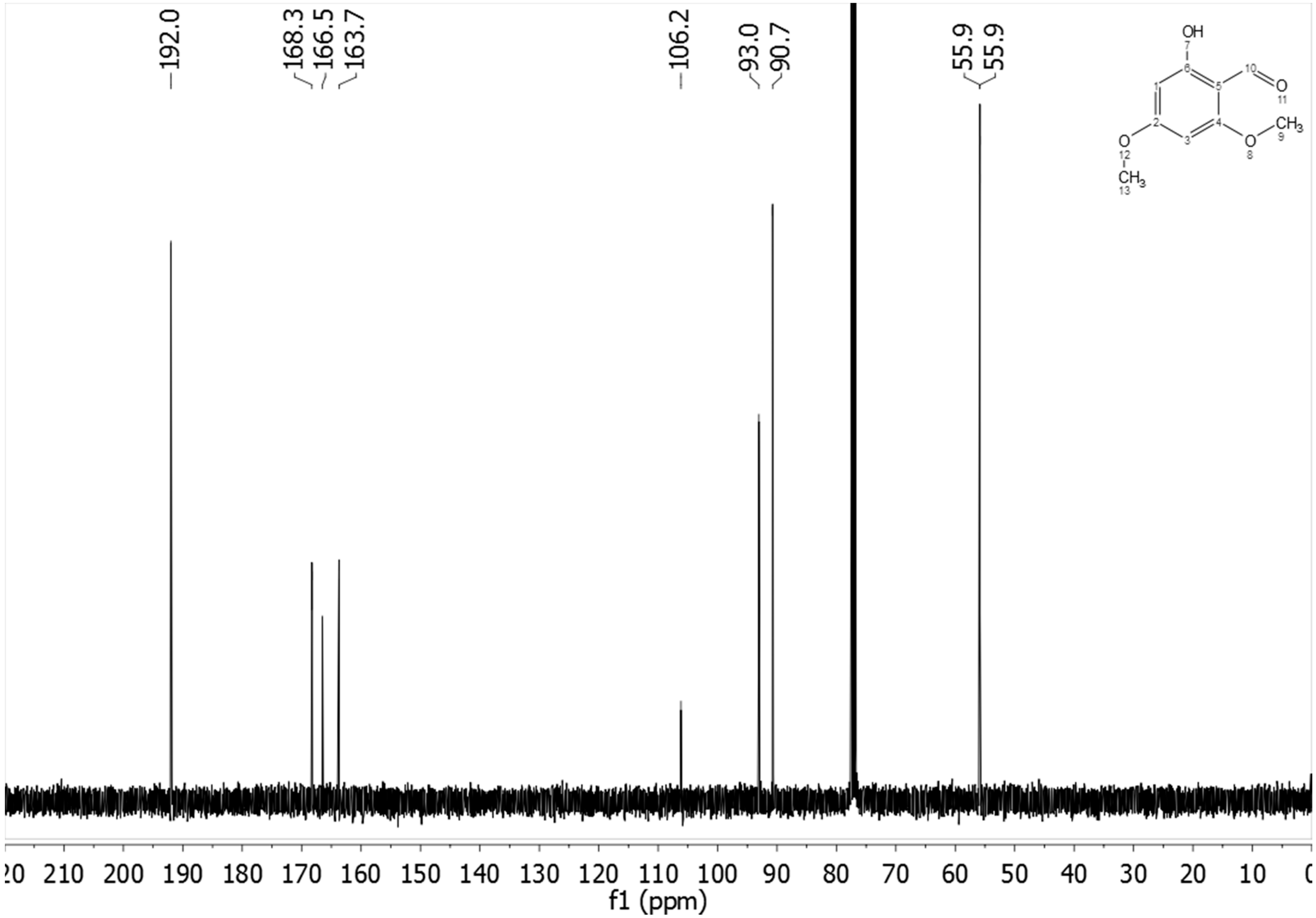

Figure S14. ^{13}C-NMR (100 MHz, $CDCl_3$): **11**.

- **Entry 6: Reaction with 3,4,5-trimethylphenol**

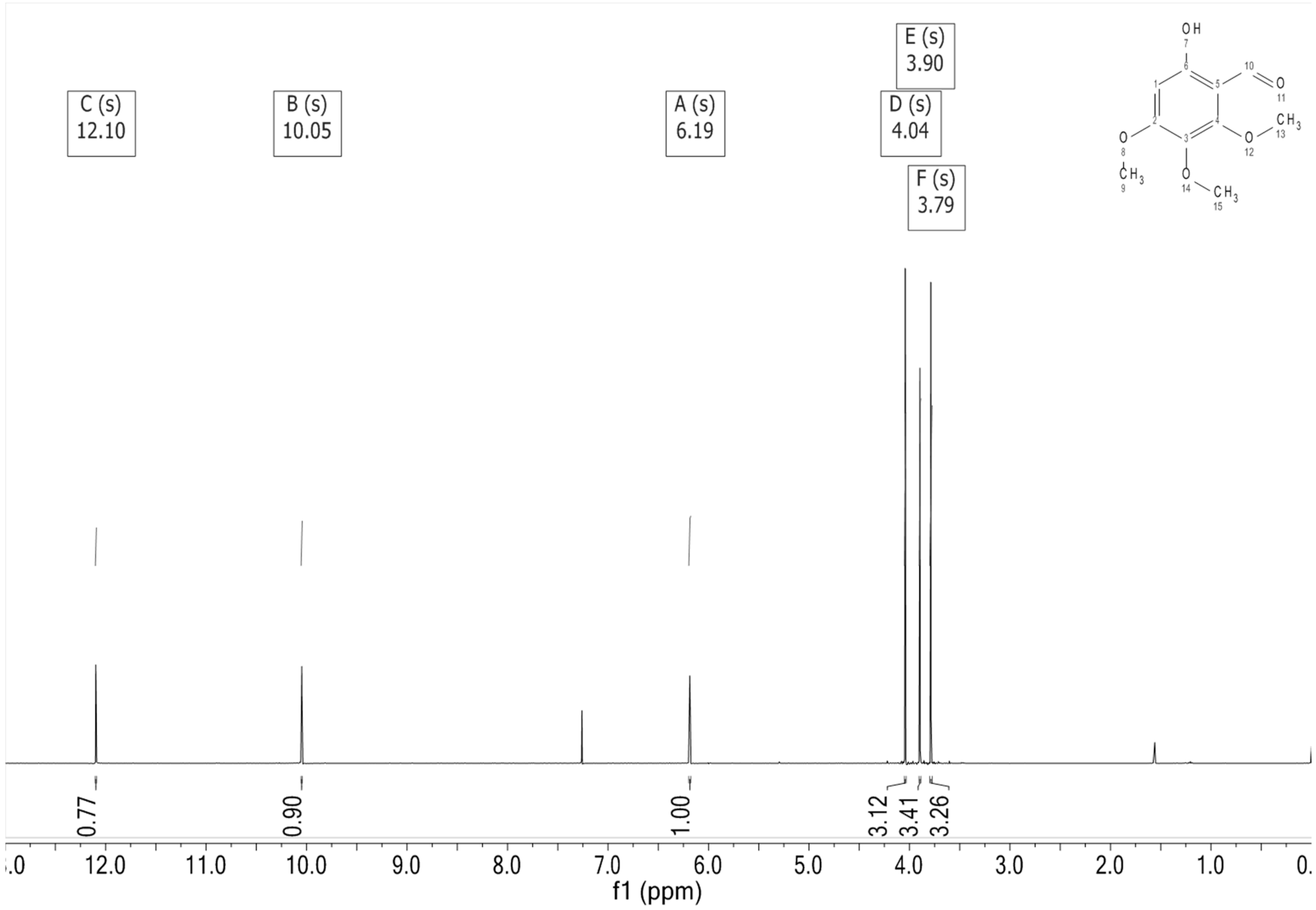

Figure S15. ^{1}H-NMR (400 MHz, $CDCl_3$): **12**.

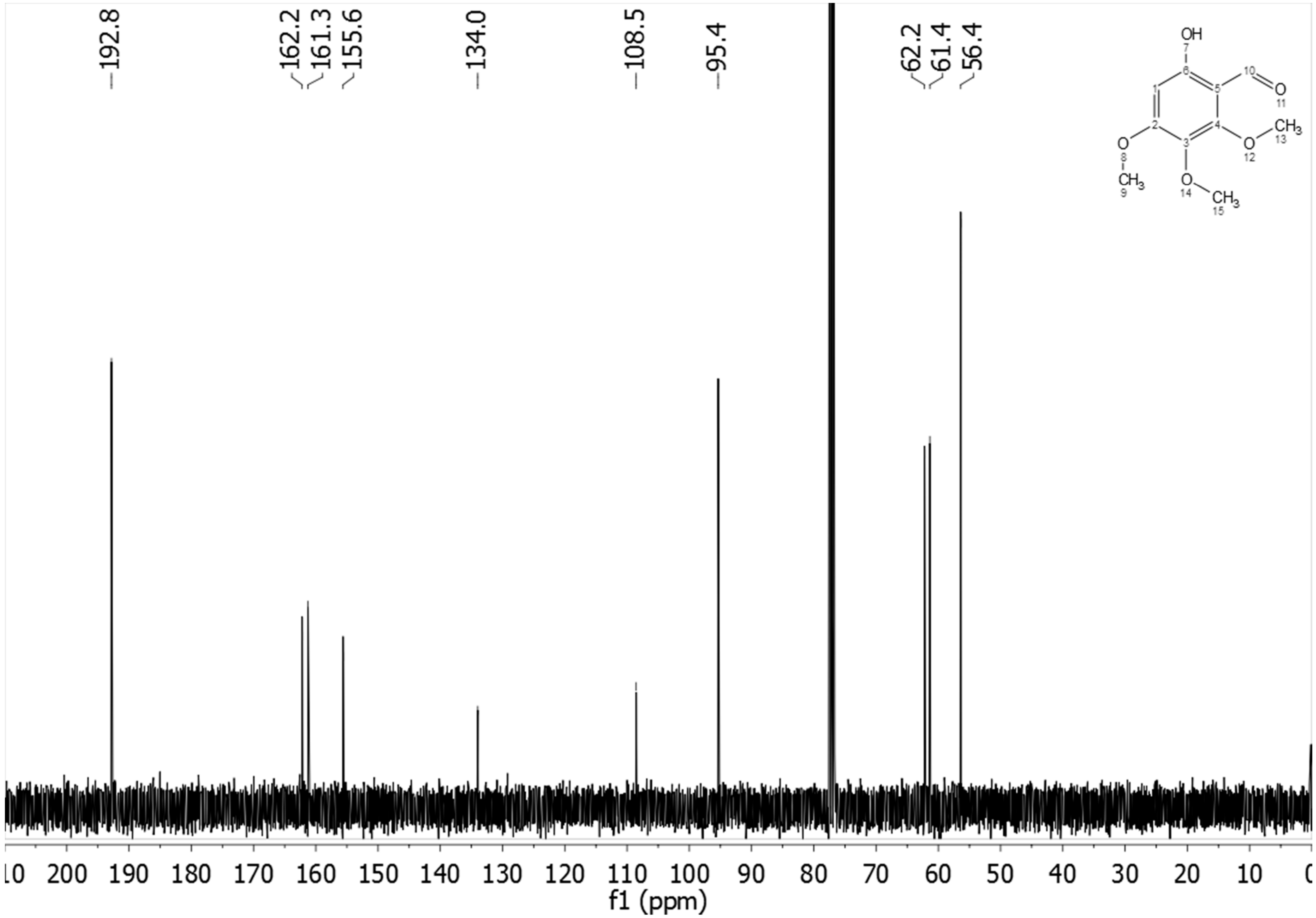

Figure S16. ^{13}C-NMR (100 MHz, $CDCl_3$): **12**.

- **Entry 7: Reaction with anisole:**

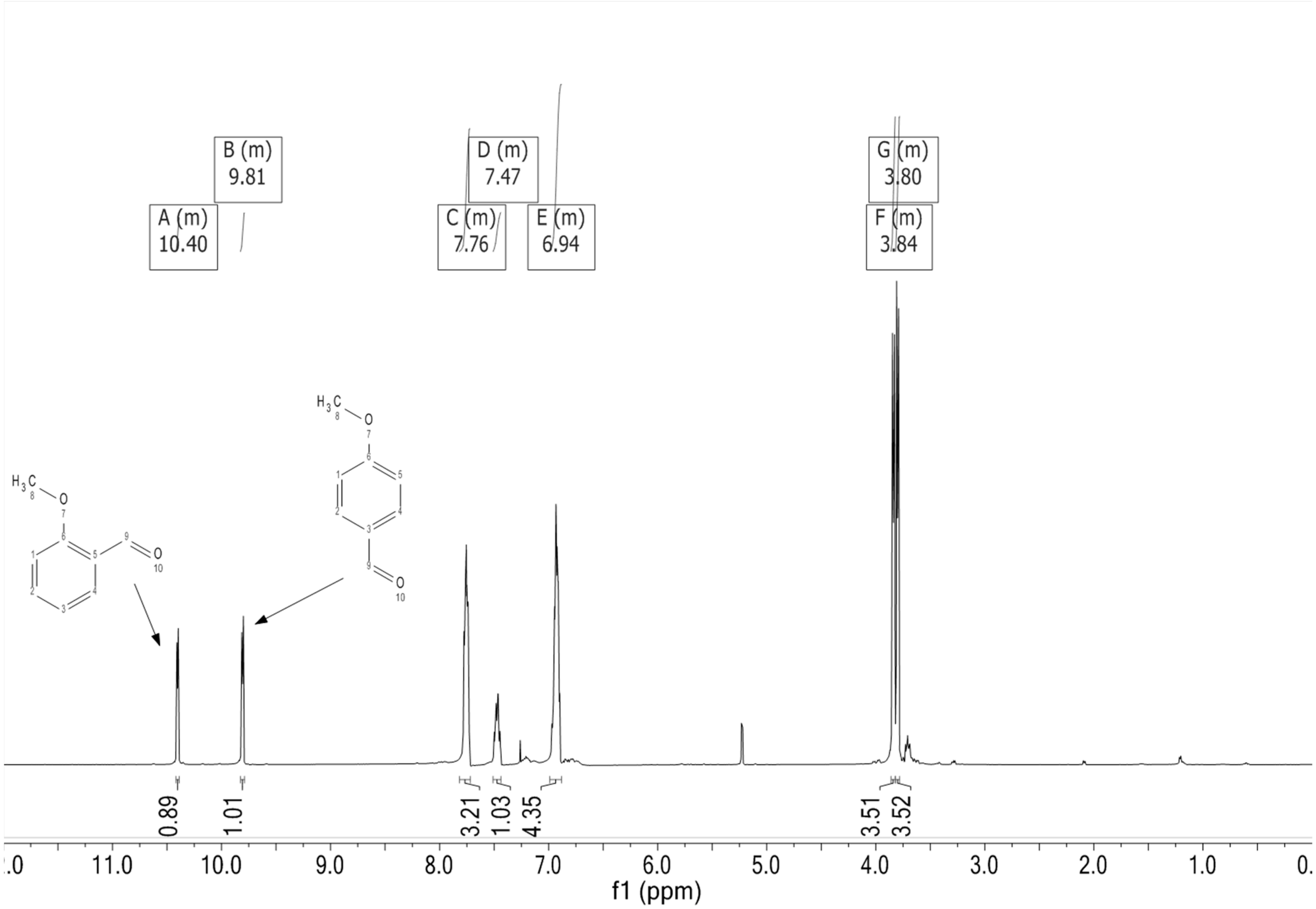

Figure S17. ^{1}H NMR (400 MHz, $CDCl_3$): **13** and **14**.

- **Entry 8: Reaction with 1,3-dimethoxybenzene:**

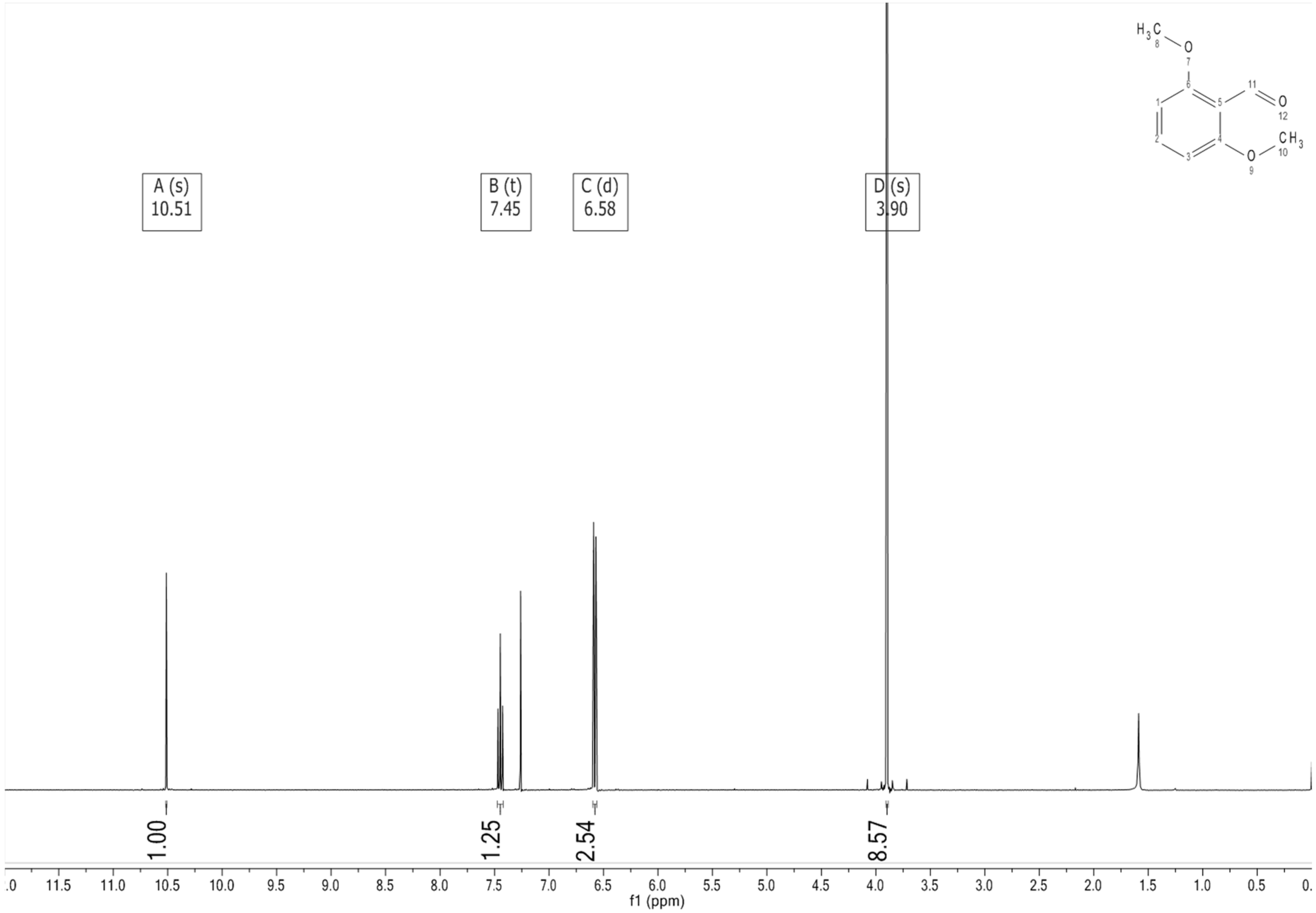

Figure S18. ^{1}H NMR (400 MHz, $CDCl_3$): **15**.

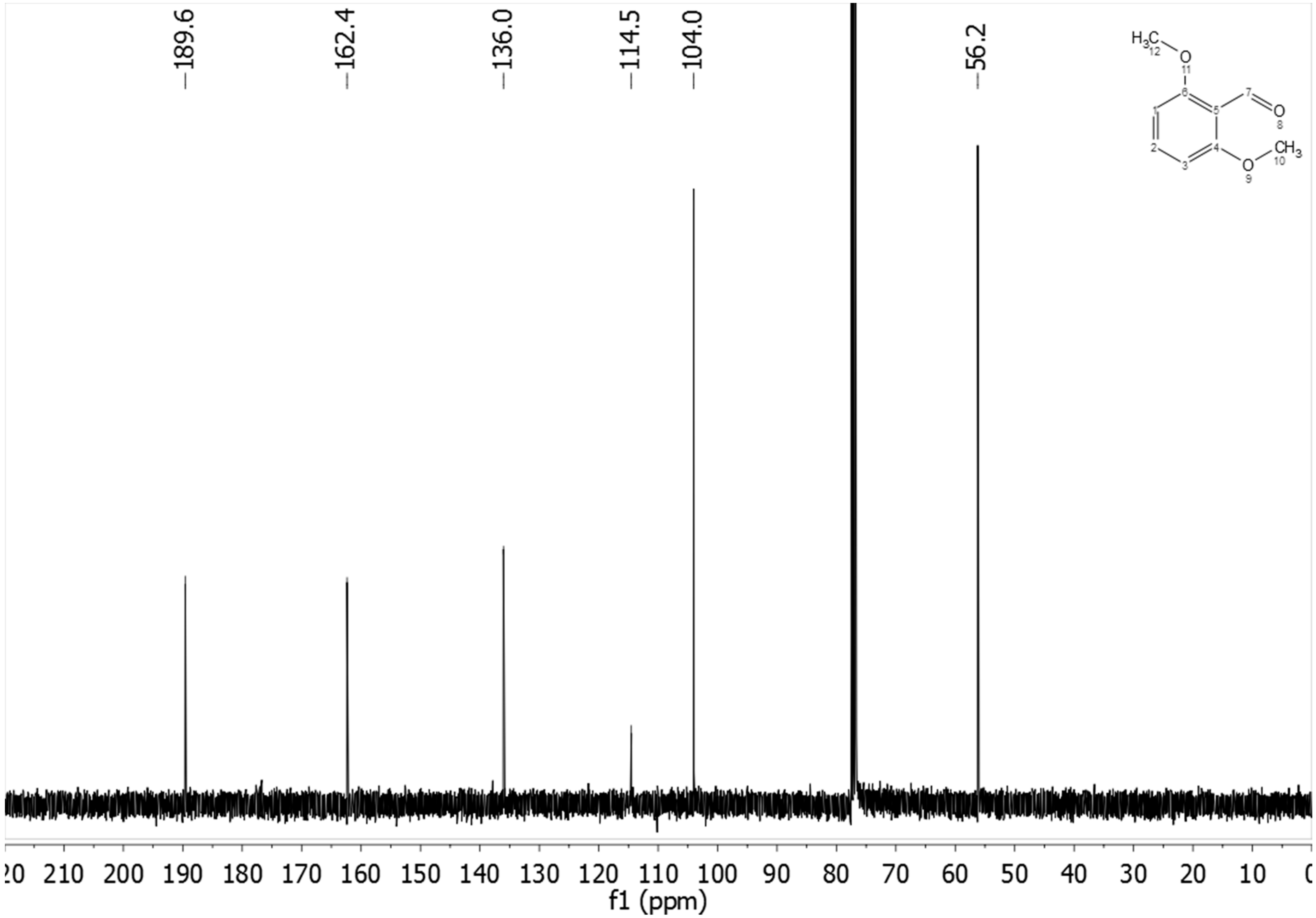

Figure S19. ^{13}C-NMR (100 MHz, $CDCl_3$): **15**.

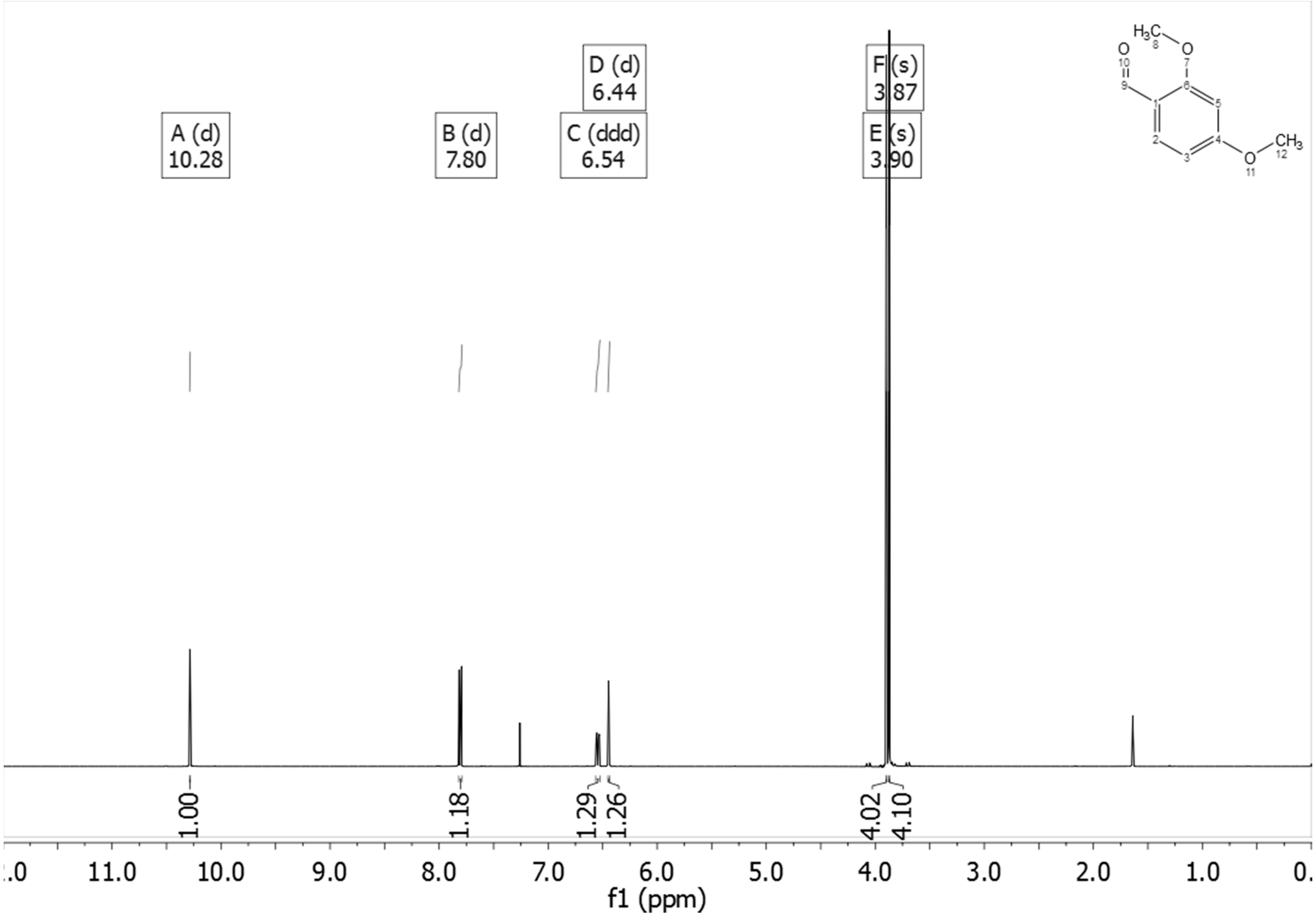

Figure S20. ^{1}H NMR (400 MHz, $CDCl_3$): **16**.

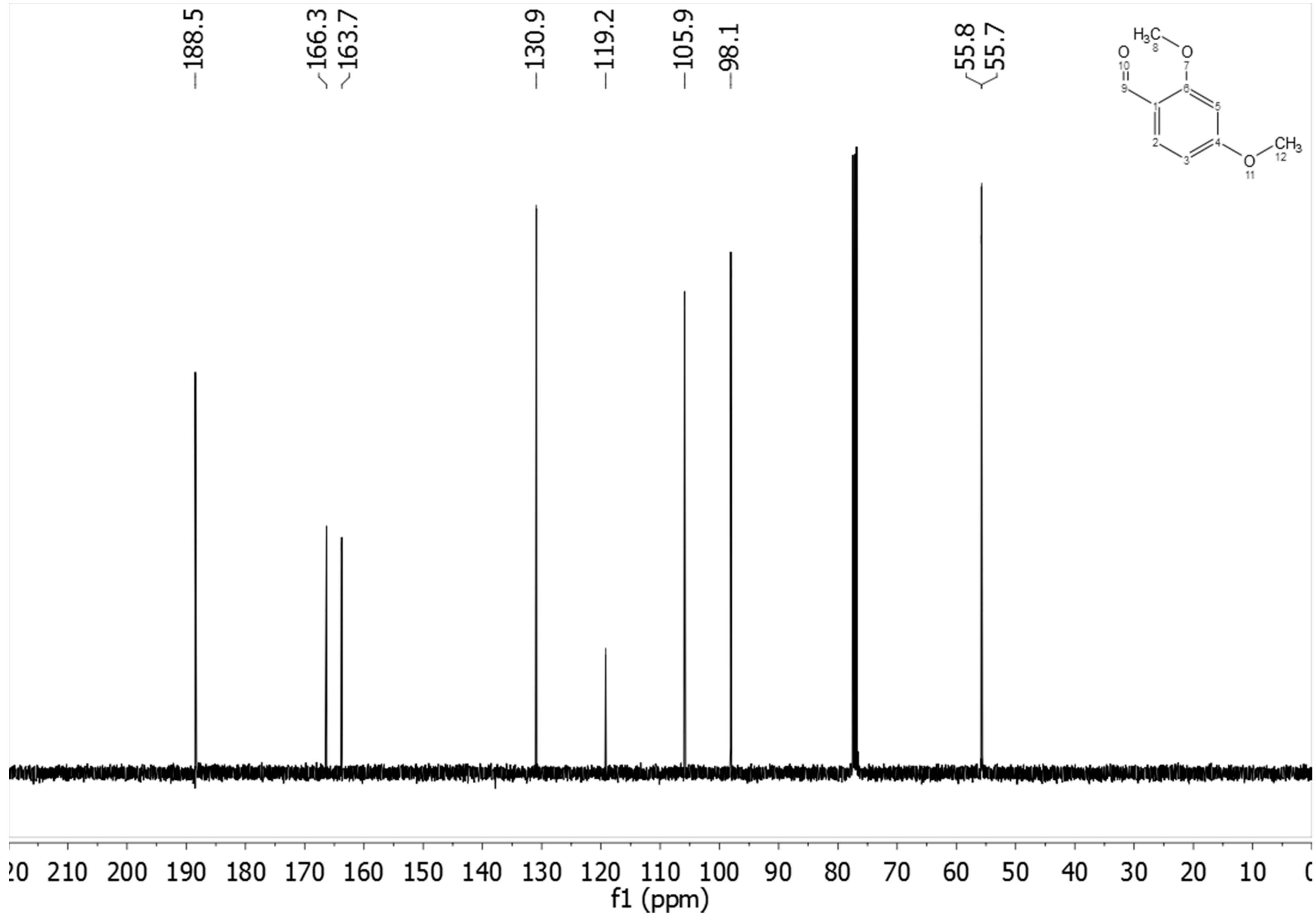

Figure S21. ^{13}C-NMR (100 MHz, $CDCl_3$): **16**.

- **Entry 9: Reaction with 1,3,5-trimethoxybenzene:**

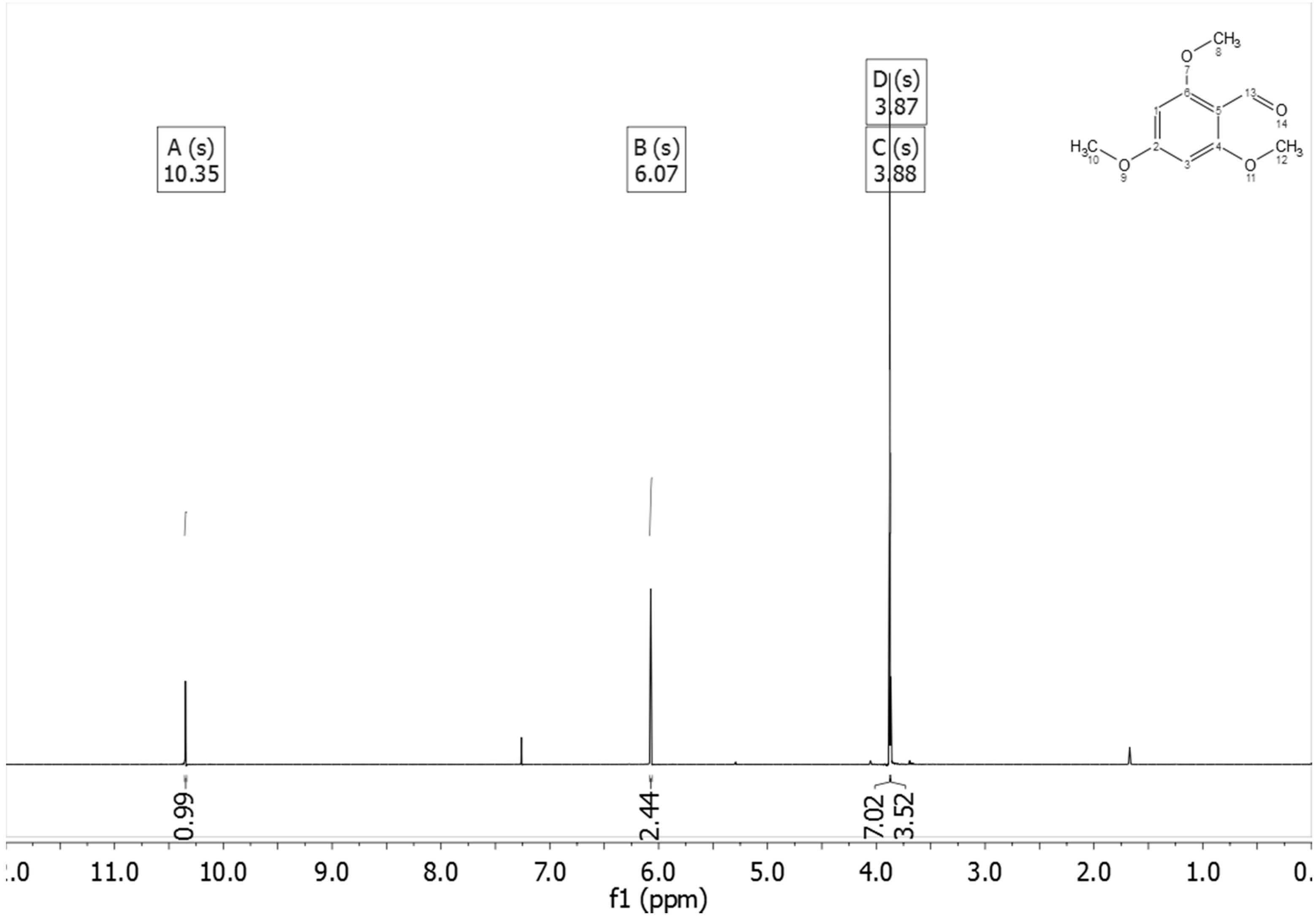

Figure S22. ^{1}H NMR (400 MHz, $CDCl_3$): **17**.

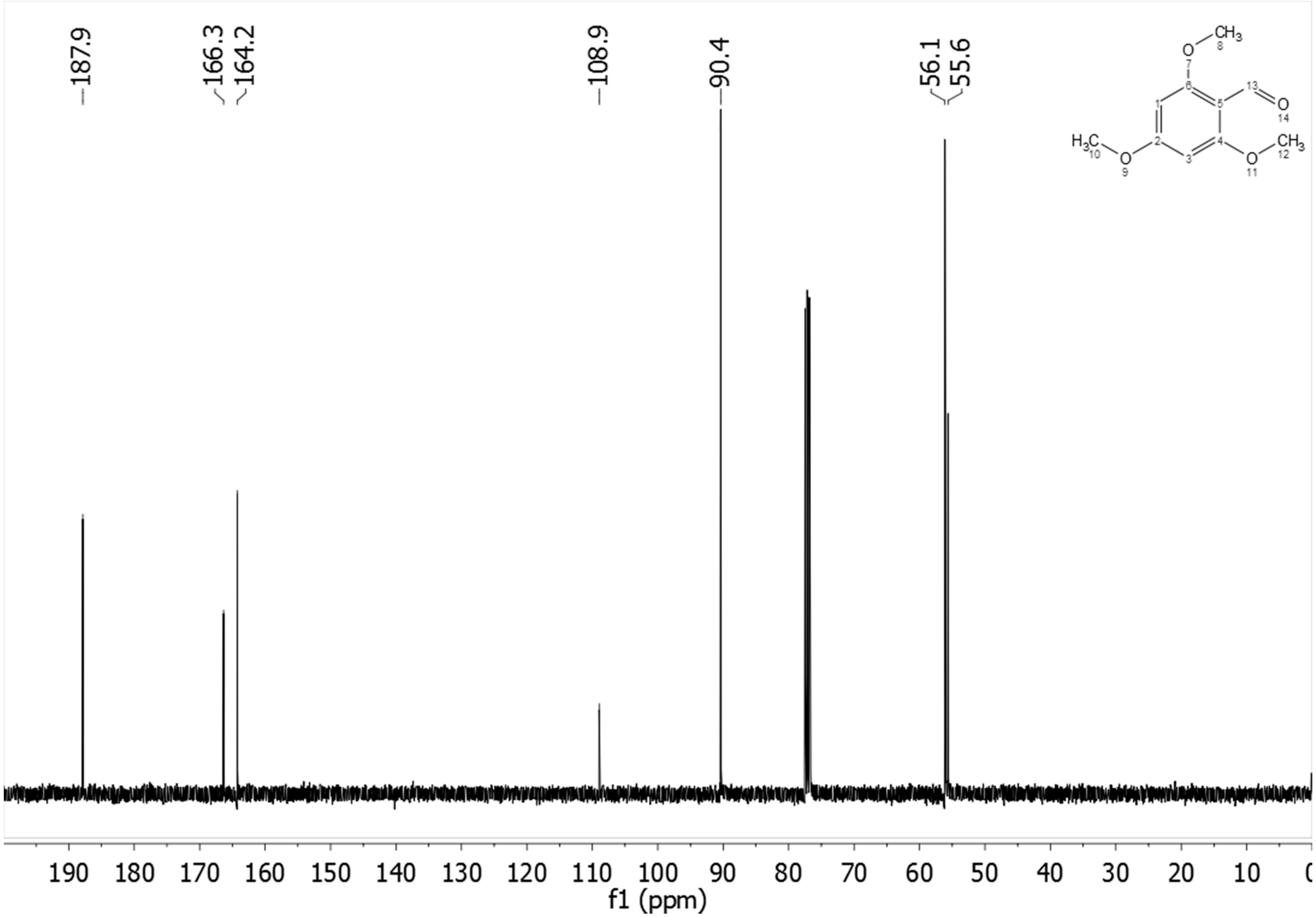

Figure S23. ^{13}C-NMR (100 MHz, $CDCl_3$): **17**.

- **Entry 10: Reaction with 3,5-dimethylanisole**

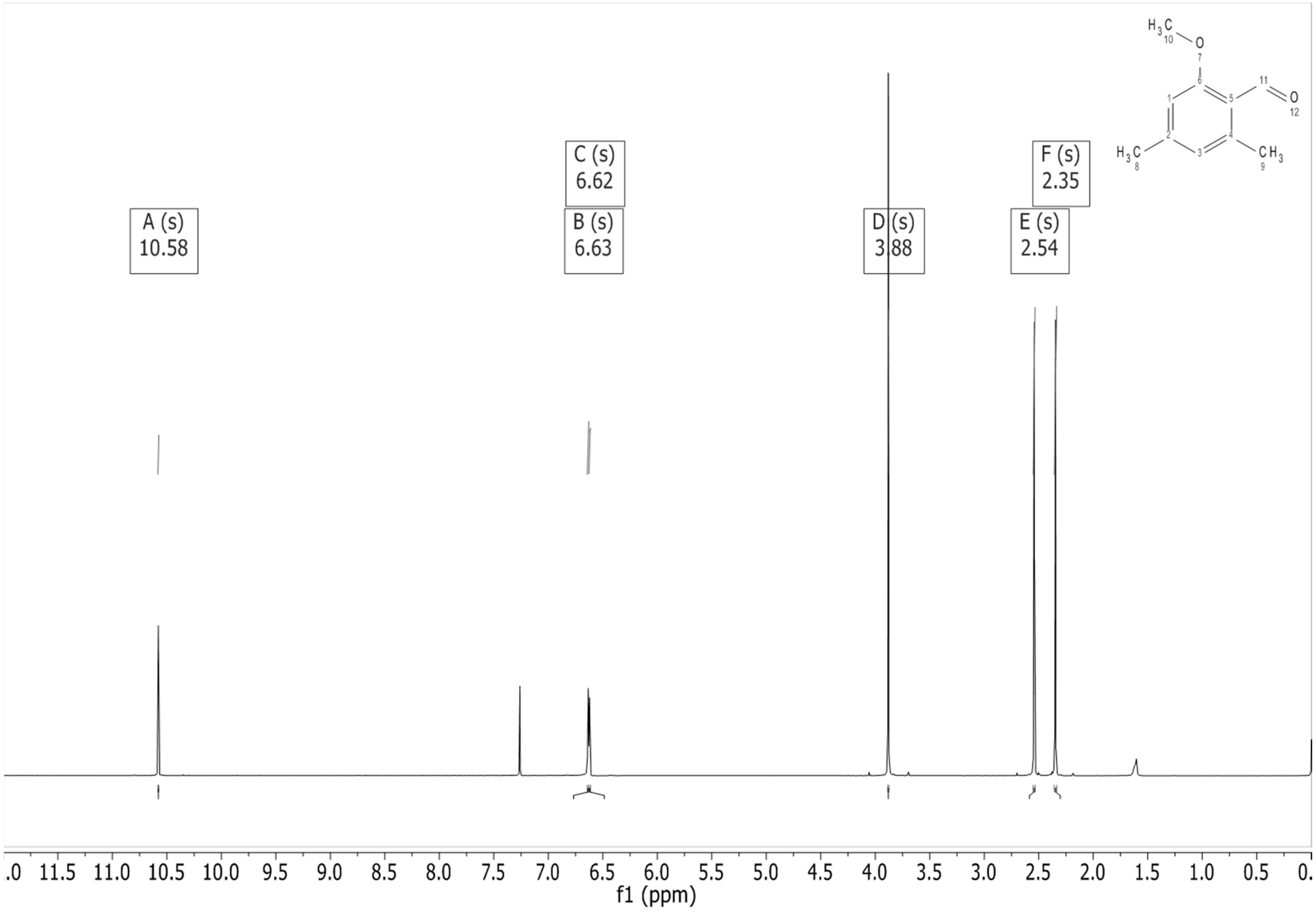

Figure S24. ^{1}H-NMR (400 MHz, $CDCl_3$): **18**.

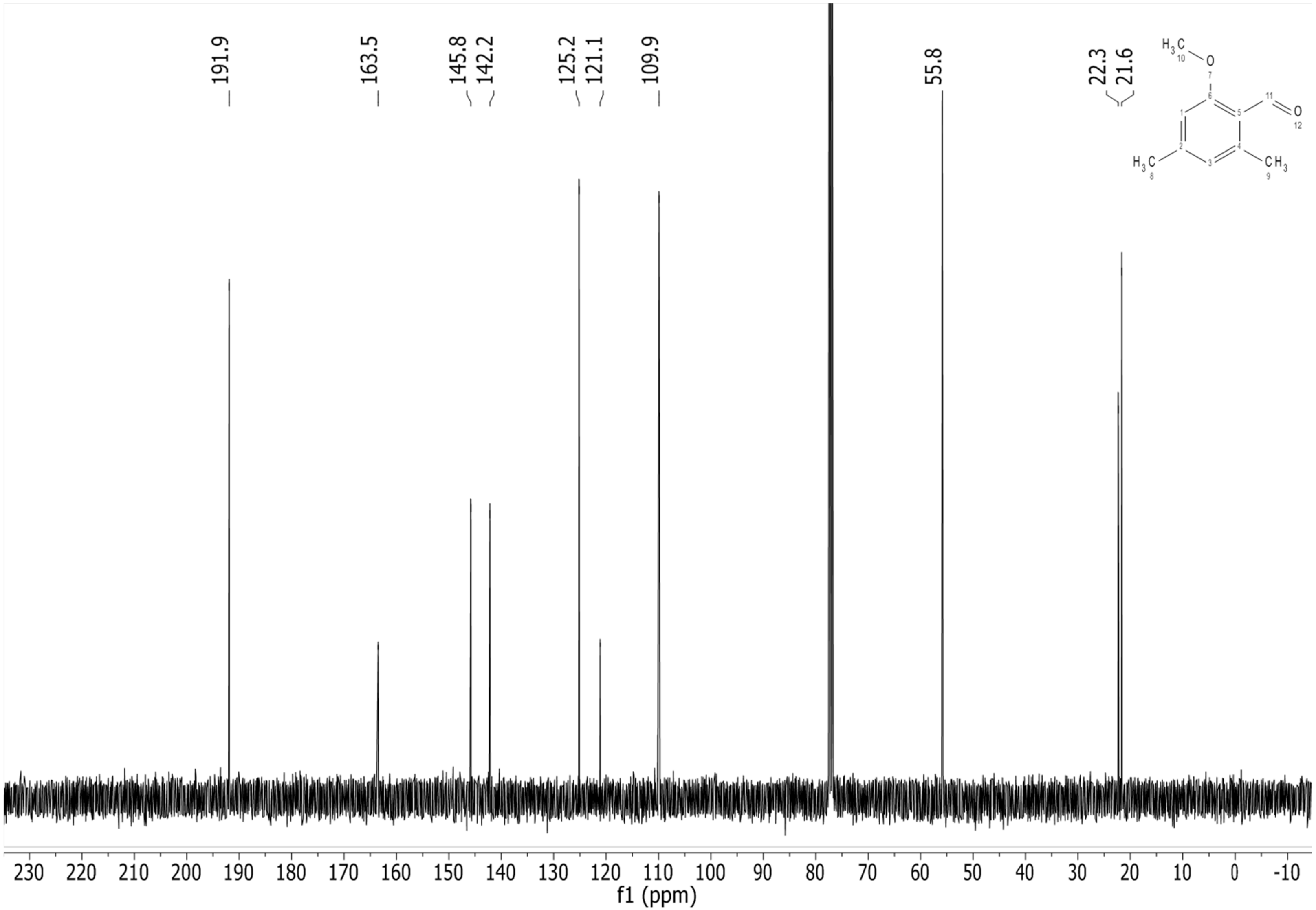

Figure S25. ^{13}C-NMR (100 MHz, $CDCl_3$): **18**.

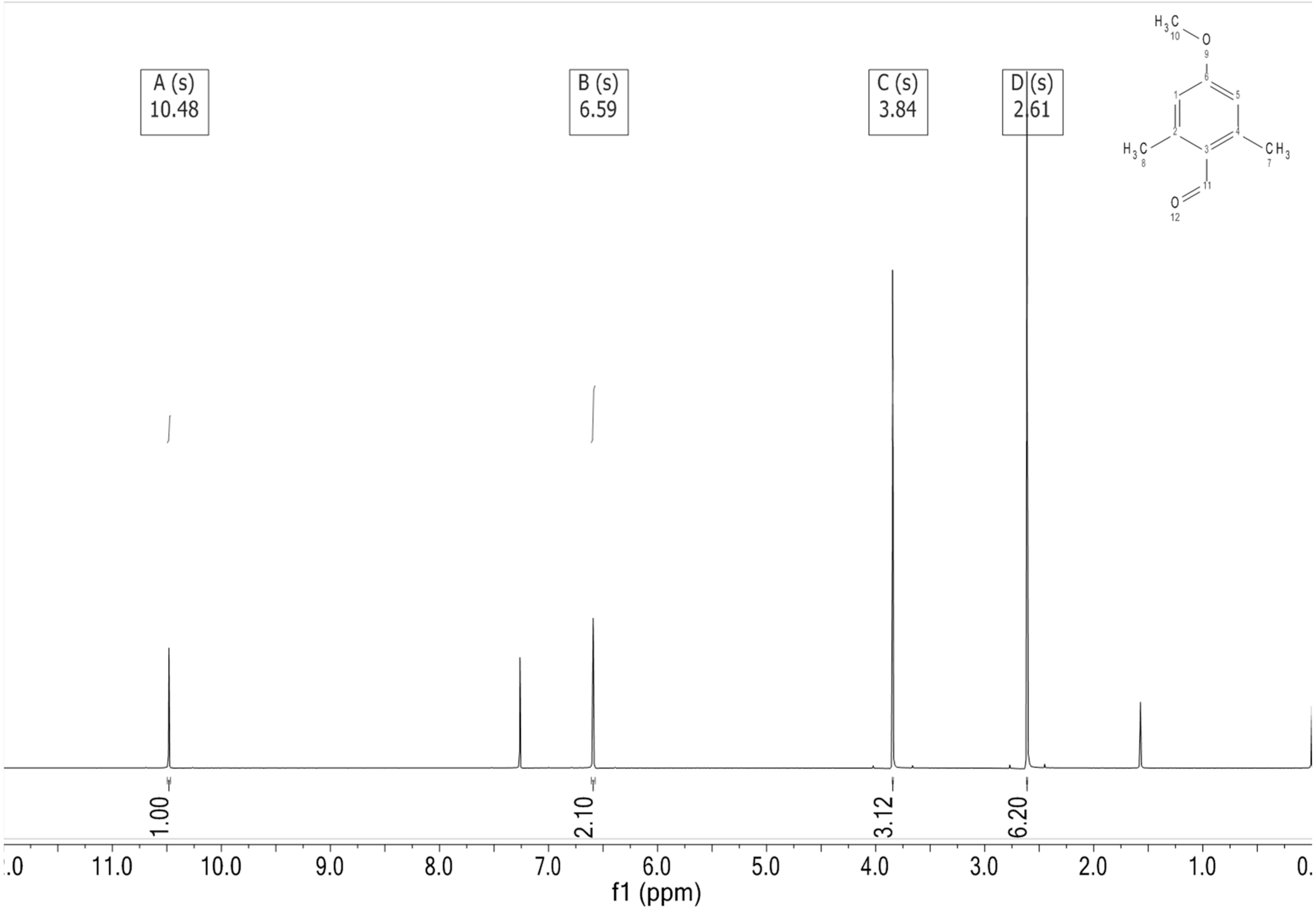

Figure S26. ^{1}H-NMR (400 MHz, $CDCl_3$): **19**.

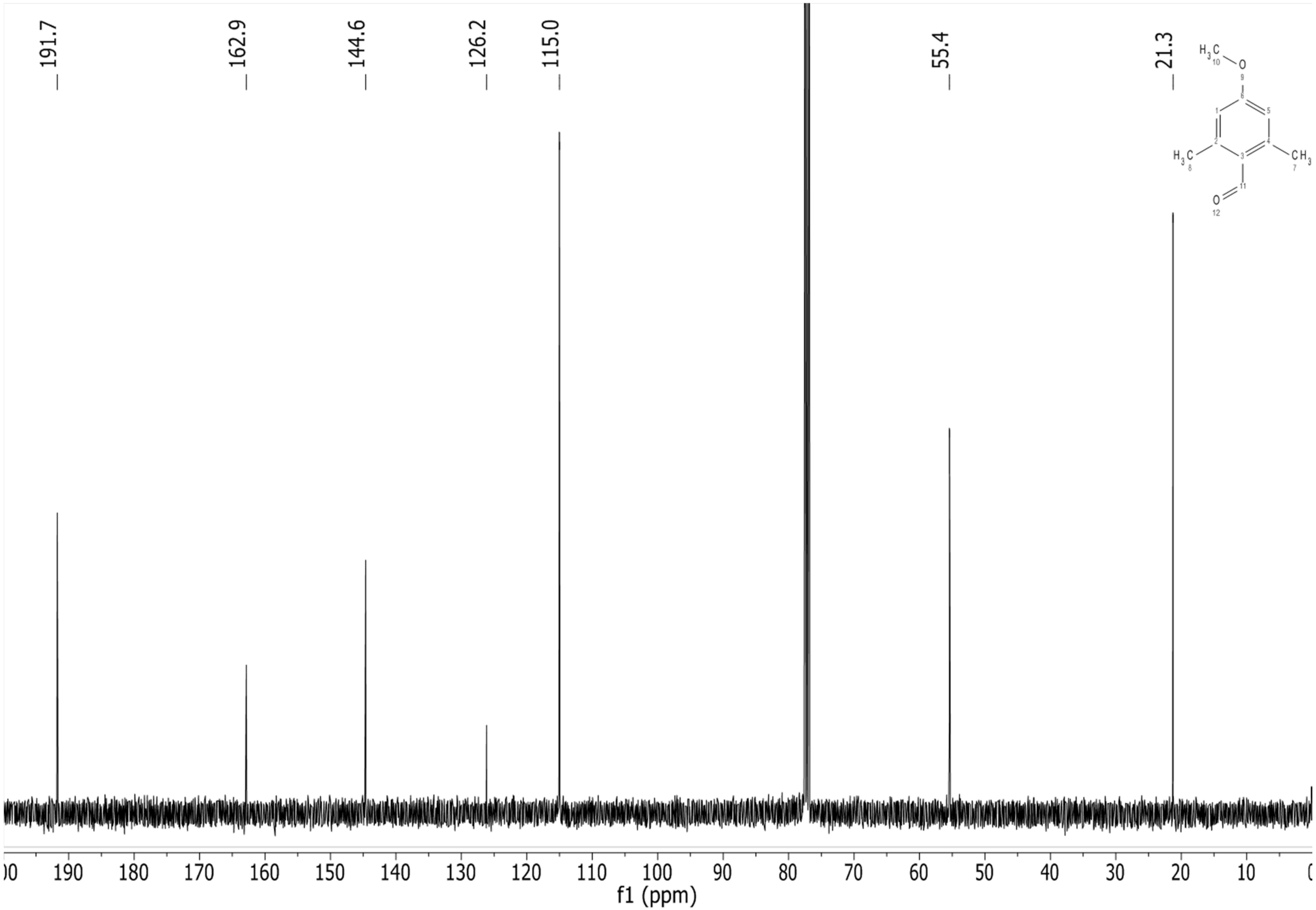

Figure S27. ^{13}C-NMR (1 01 MHz, $CDCl_3$): **19**.

- **Entry 11: Reaction with o-xylene:**

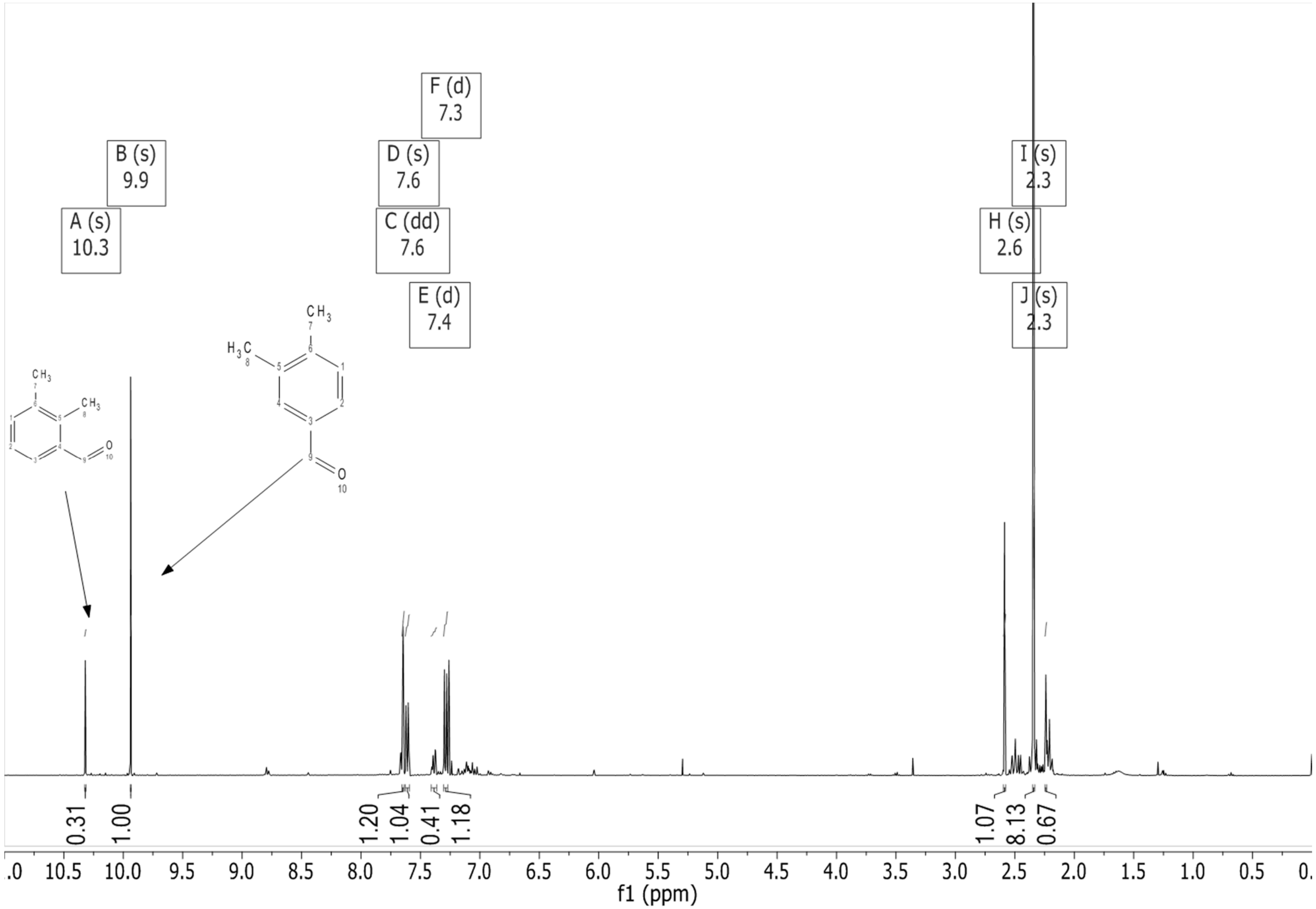

Figure S28. ^{1}H-NMR (400 MHz, $CDCl_3$): **20** and **21**.

- **Entry 12: Reaction with *m*-xylene**

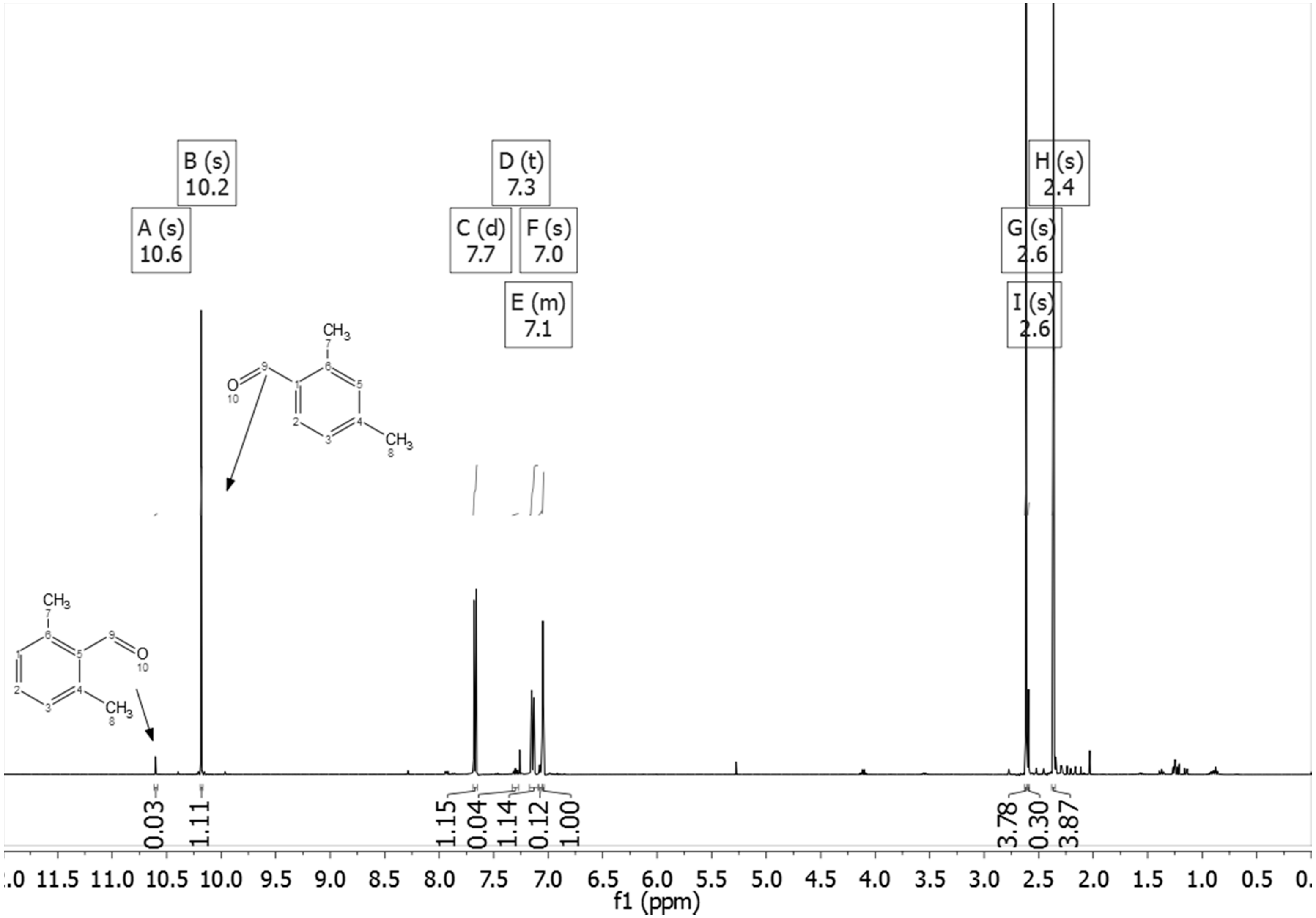

Figure S29. ^{1}H-NMR (400 MHz, $CDCl_3$): **22** and **23**.

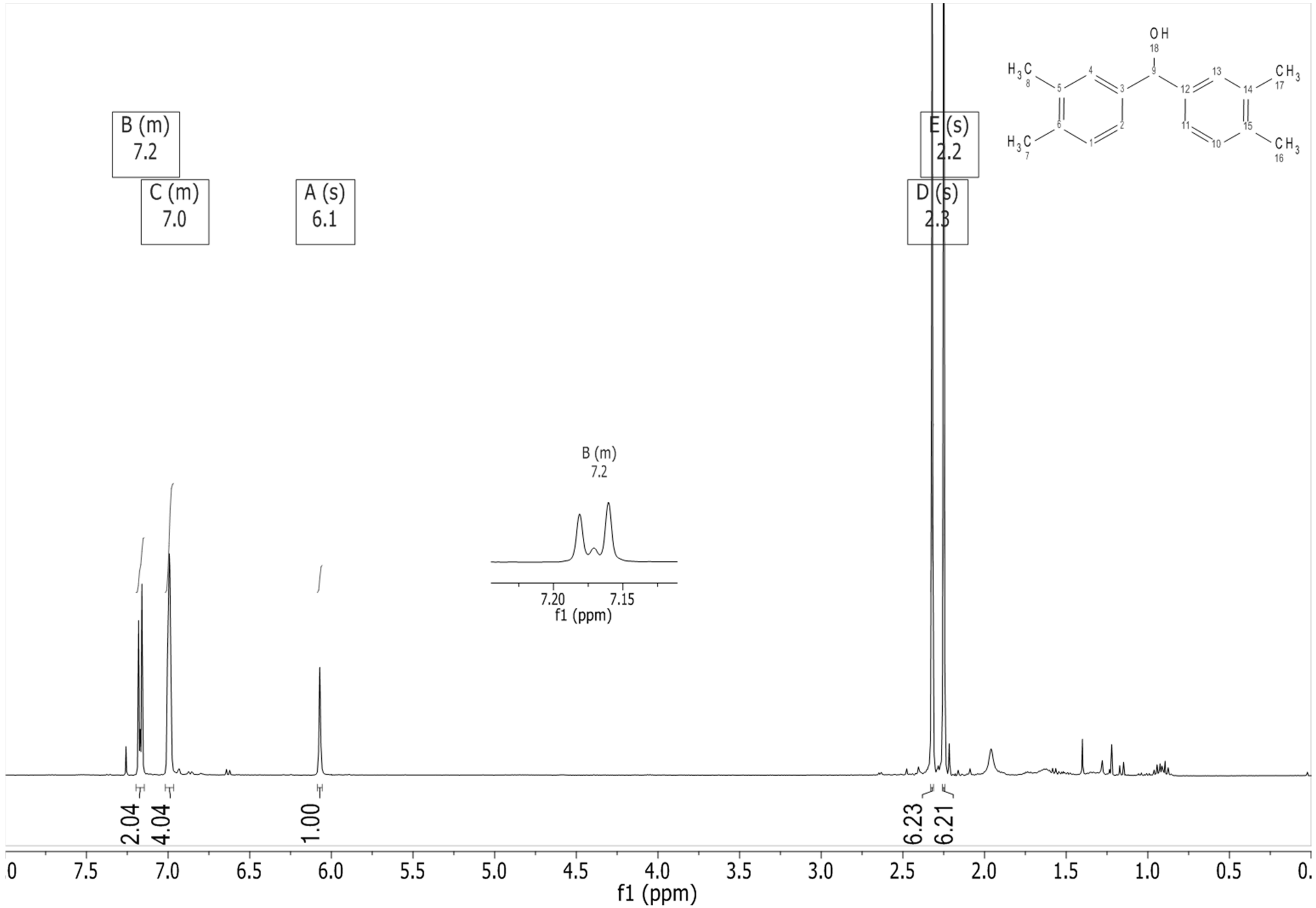

Figure S30. ^{1}H-NMR (400 MHz, $CDCl_3$): **24**.

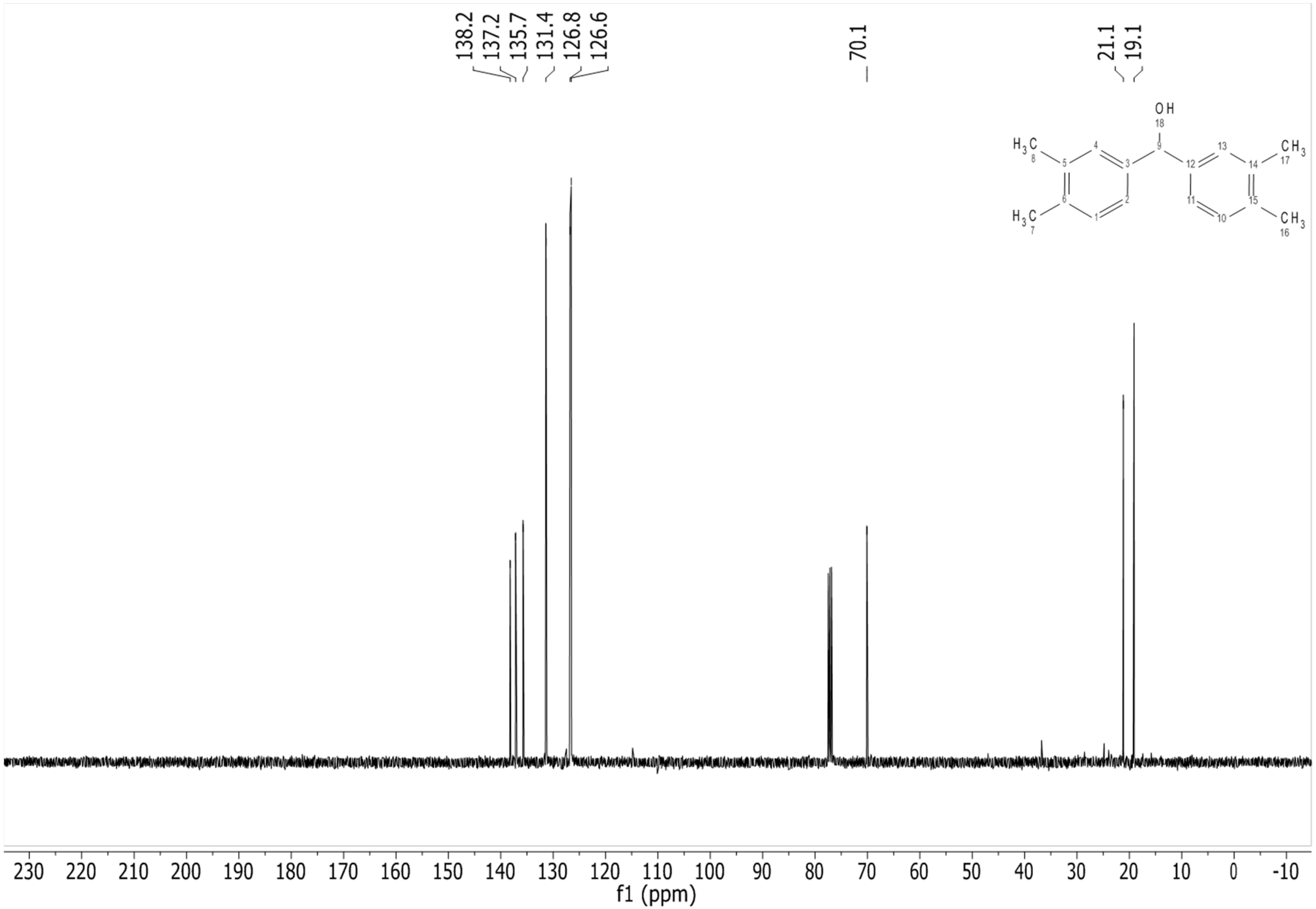

Figure S31. ^{13}C-NMR (1 01 MHz, $CDCl_3$): **24**.

➢ **Entry 13: Reaction with *p*-xylene:**

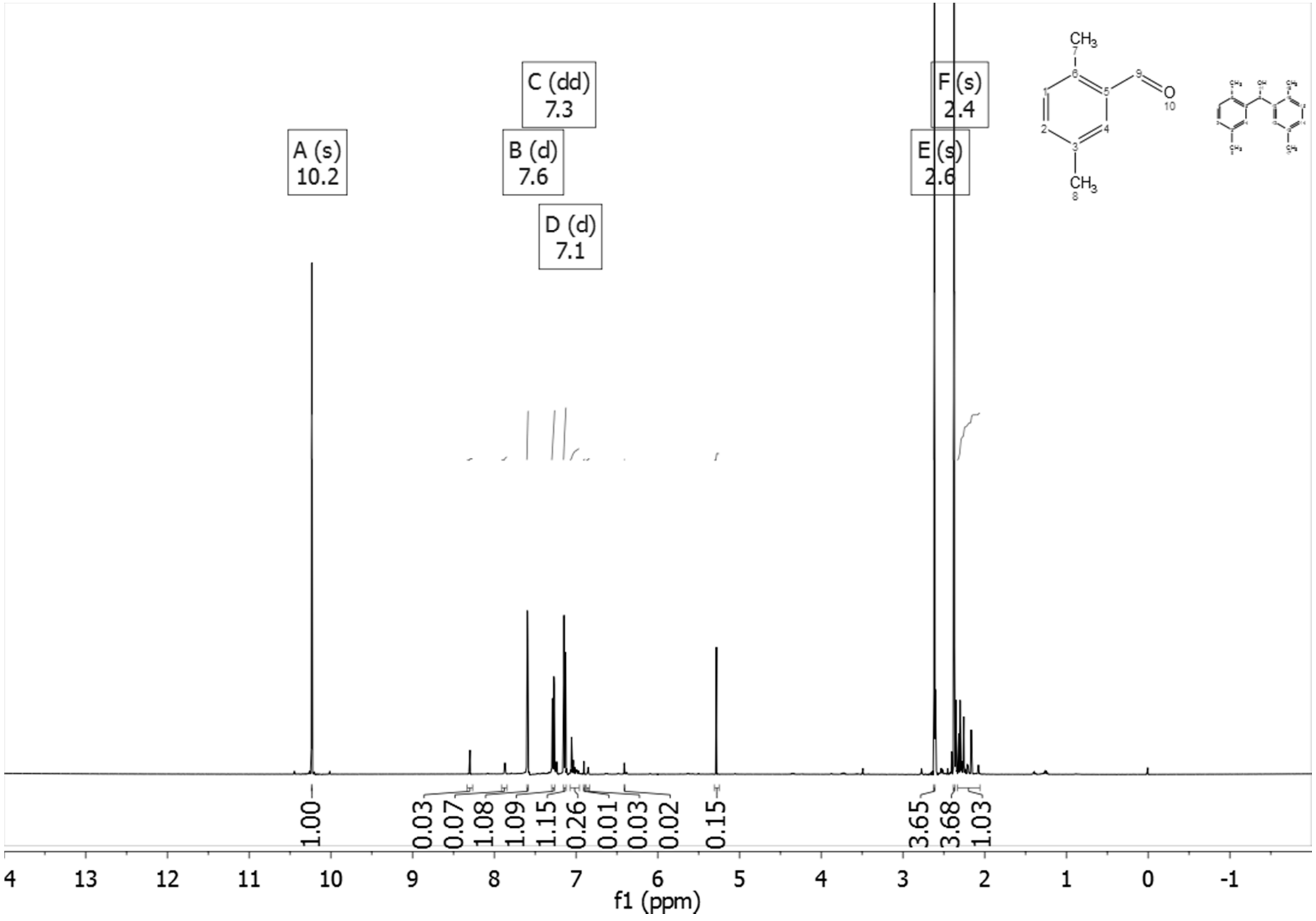

Figure S32. ^{1}H-NMR (400 MHz, $CDCl_3$): **25** and byproduct.

- **Entry 14: Reaction with mesytilene:**

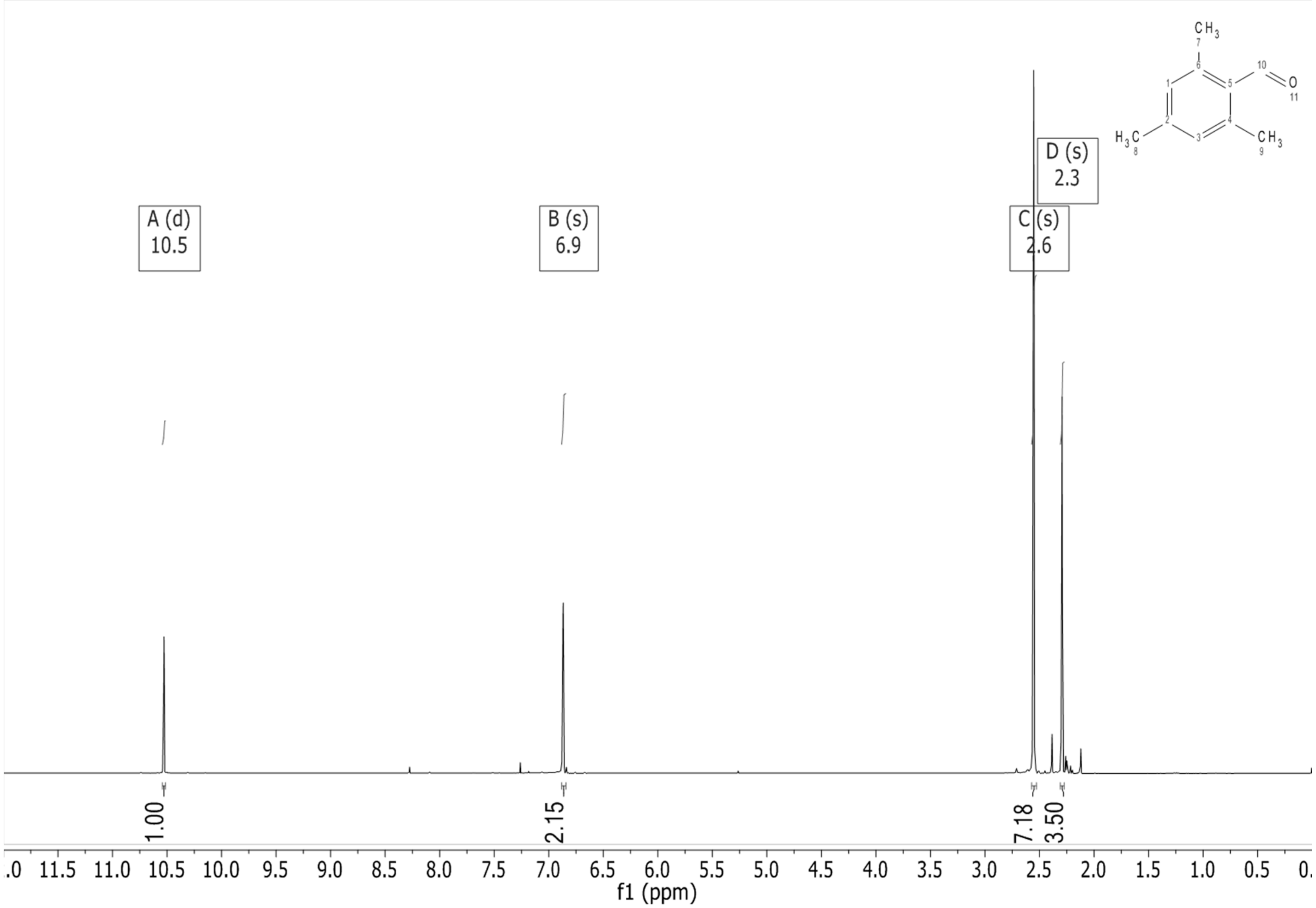

Figure 33. ^{1}H-NMR (400 MHz, $CDCl_3$): **26**.

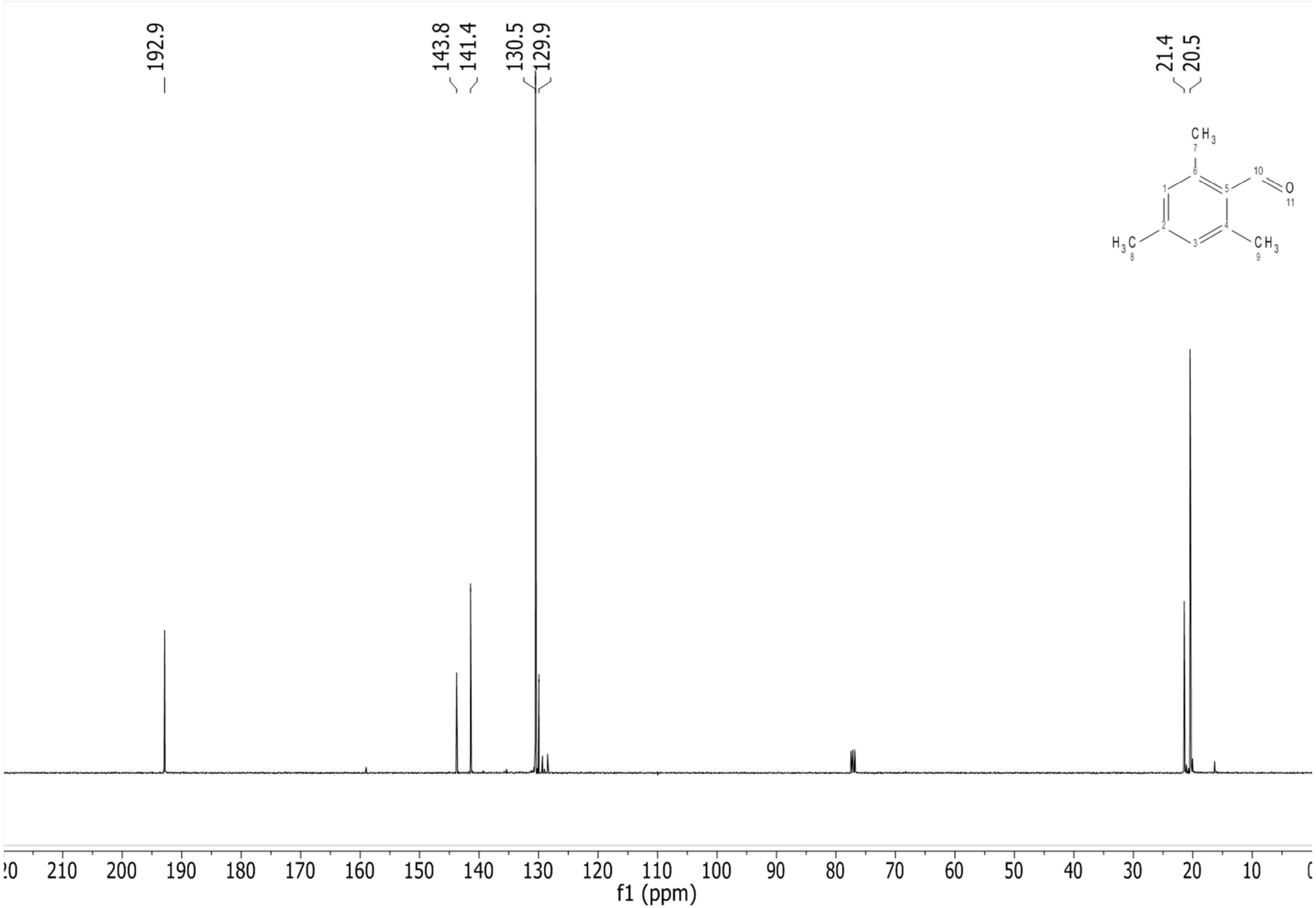

Figure S34. ^{13}C-NMR (100 MHz, $CDCl_3$): **26**.

2. High performance liquid chromatography (HPLC): Reaction crudes

- **Entry 1: Reaction with phenol**

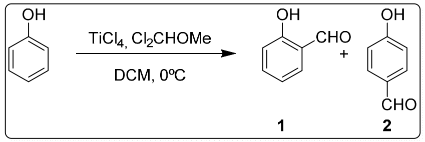

HPLC crude:

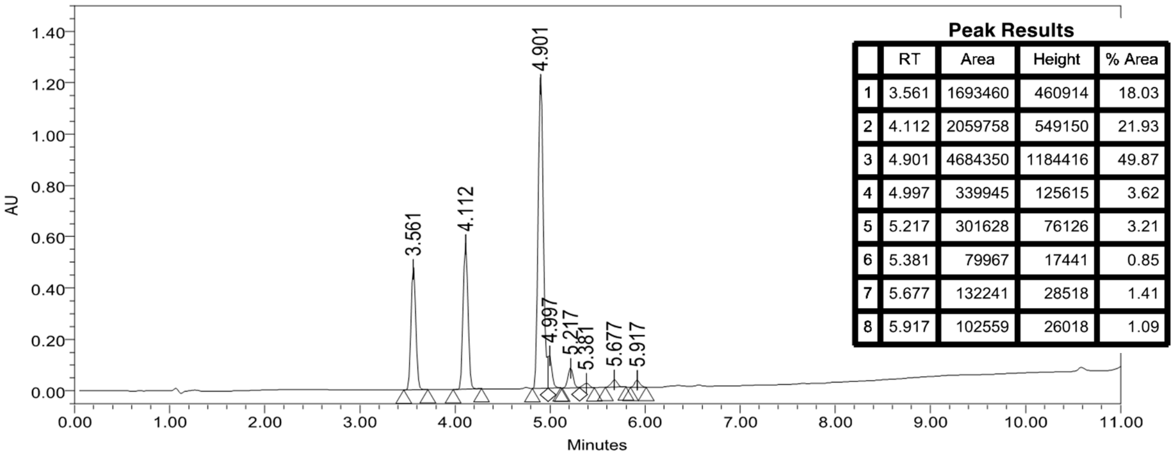

	RT	Area	Height	% Area
1	3.561	1693460	460914	18.03
2	4.112	2059758	549150	21.93
3	4.901	4684350	1184416	49.87
4	4.997	339945	125615	3.62
5	5.217	301628	76126	3.21
6	5.381	79967	17441	0.85
7	5.677	132241	28518	1.41
8	5.917	102559	26018	1.09

t_R = 3.56 min → 4-hydroxybenzaldehyde (2); t_R = 4.11 min → phenol (starting material); t_R = 4.90 min → 2-hydroxybenzaldehyde(1)

UV-Vis:

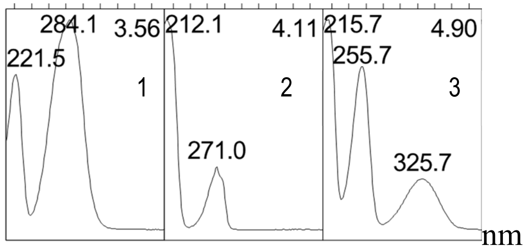

Entry 2: Reaction with 3-methylphenol

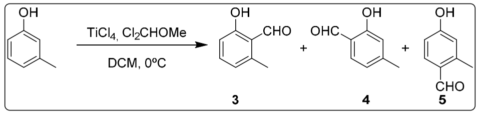

HPLC crude:

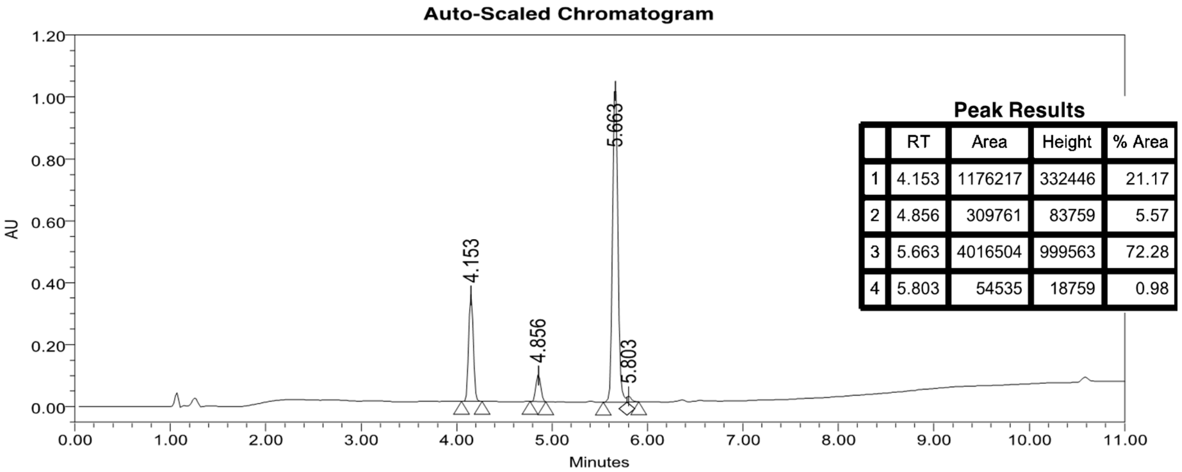

	RT	Area	Height	% Area
1	4.153	1176217	332446	21.17
2	4.856	309761	83759	5.57
3	5.663	4016504	999563	72.28
4	5.803	54535	18759	0.98

t_R = 4.15 min → 4-hydroxy-2-methylbenzaldehyde (5); t_R = 4.86 min → 3-methylphenol (starting material); t_R = 5.66 min → 2-hydroxy-6-methylbenzaldehyde (3) + 2-hydroxy-4-methylbenzaldehyde (4).

UV-VIS:

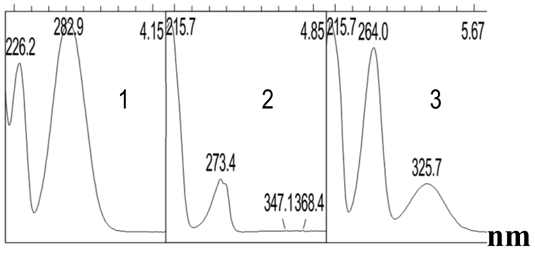

➢ Entry 3: Reaction with 3-methoxyphenol:

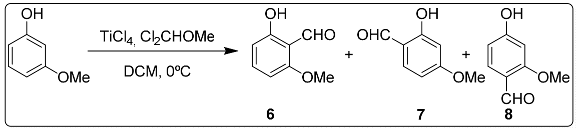

HPLC crude:

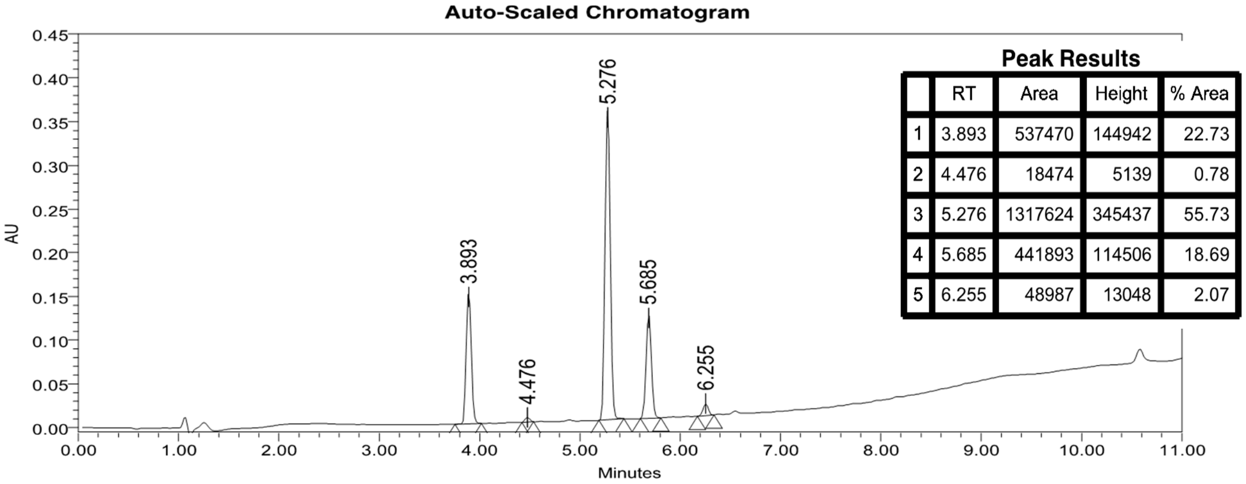

Peak Results

	RT	Area	Height	% Area
1	3.893	537470	144942	22.73
2	4.476	18474	5139	0.78
3	5.276	1317624	345437	55.73
4	5.685	441893	114506	18.69
5	6.255	48987	13048	2.07

t_R = 3.89 min →4-hydroxy-2-methoxylbenzaldehyde (8); t_R = 5.28 min → 2-hydroxy-4-methoxybenzaldehyde (7); t_R = 5.69 min → 2-hydroxy-6-methoxybenzaldehyde (6).

UV-VIS:

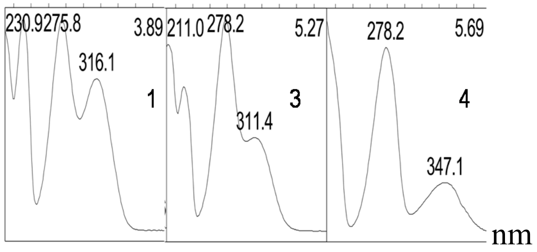

➢ Entry 4: Reaction with 3,5-dimethylphenol:

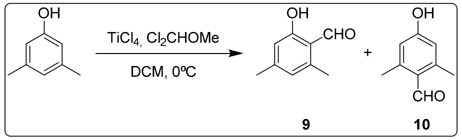

HPLC crude:

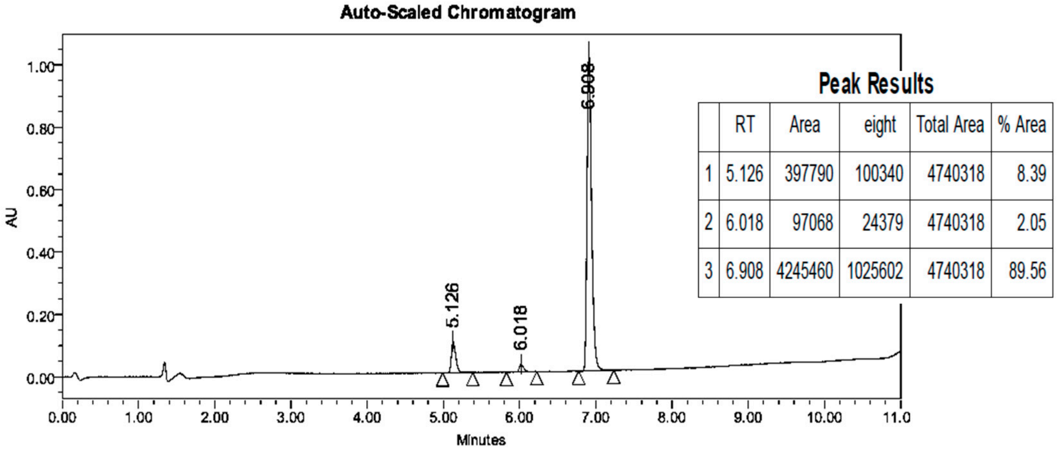

Peak Results

	RT	Area	eight	Total Area	% Area
1	5.126	397790	100340	4740318	8.39
2	6.018	97068	24379	4740318	2.05
3	6.908	4245460	1025602	4740318	89.56

t_R = 5.13 min → 4-hydroxy-2,6-dimethylbenzaldehyde (10); t_R = 6.02 min → 3,5-dimethylphenol (starting material); t_R = 6.91 min → 2-hydroxy-4,6-dimethylbenzaldehyde (9).

UV-VIS:

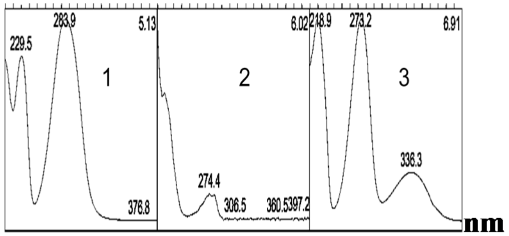

Entry 5: Reaction with 3,5-dimethoxyphenol:

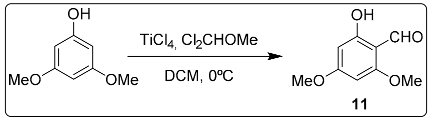

HPLC crude:

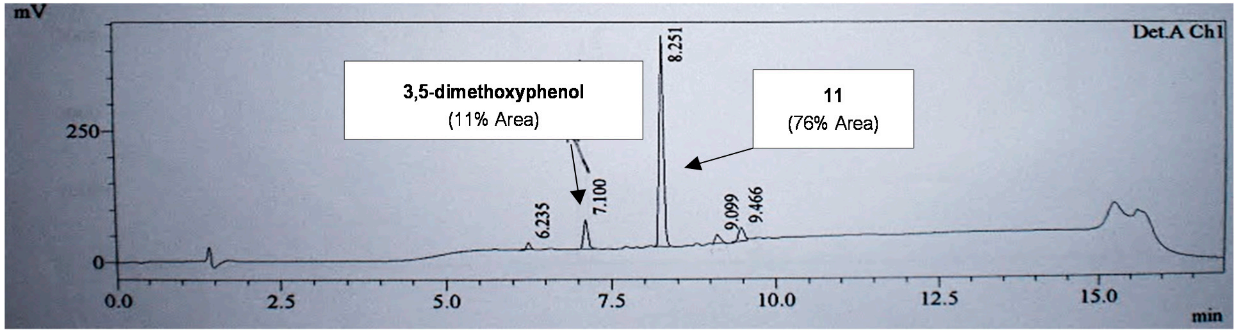

HPLC B* (X-Bridge, C_{18}, 5-100% ACN, t = 11 min)

➢ **Entry 6: Reaction with 3,4,5-trimethoxyphenol:**

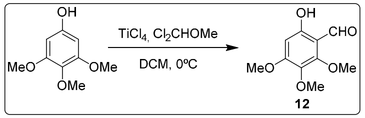

HPLC crude:

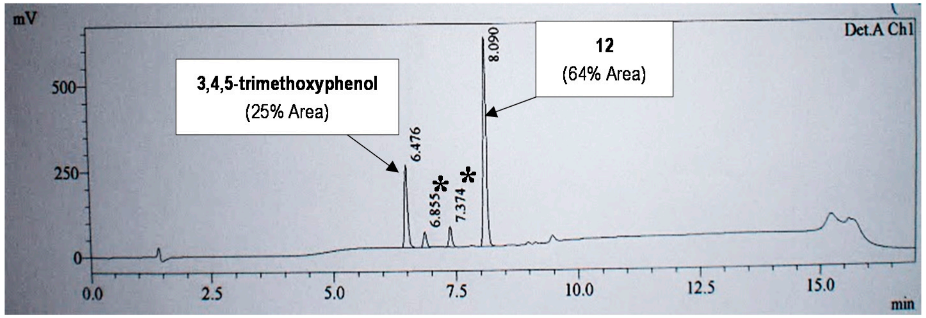

* Impurities from the starting material. HPLC B* (X-Bridge, C_{18}, 5-100% ACN, t = 11 min).

➢ Entry 7: Reaction with anisole:

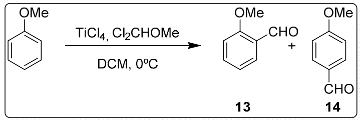

HPLC Crude:

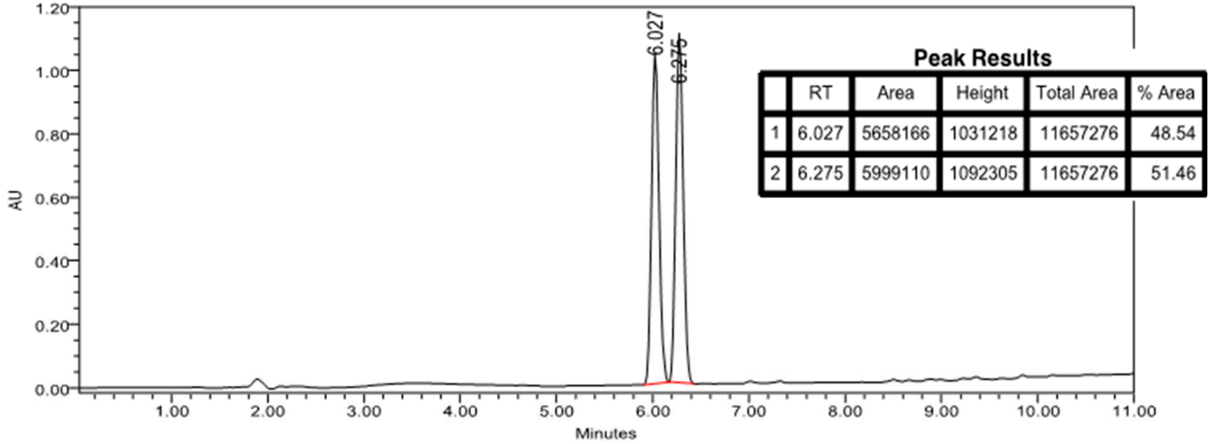

Peak Results

	RT	Area	Height	Total Area	% Area
1	6.027	5658166	1031218	11657276	48.54
2	6.275	5999110	1092305	11657276	51.46

t_R = 6.03 min → 2-methoxybenzaldehyde (**13**); t_R = 6.28 min → 4-methoxybenzaldehyde (**14**).

UV-Vis:

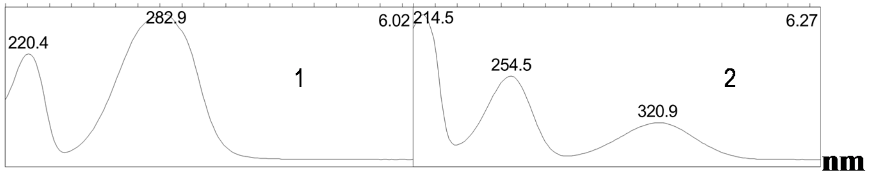

- **Entry 8: Reaction with 1,3-dimethoxybenzene:**

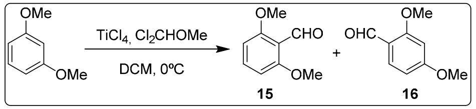

HPLC Crude:

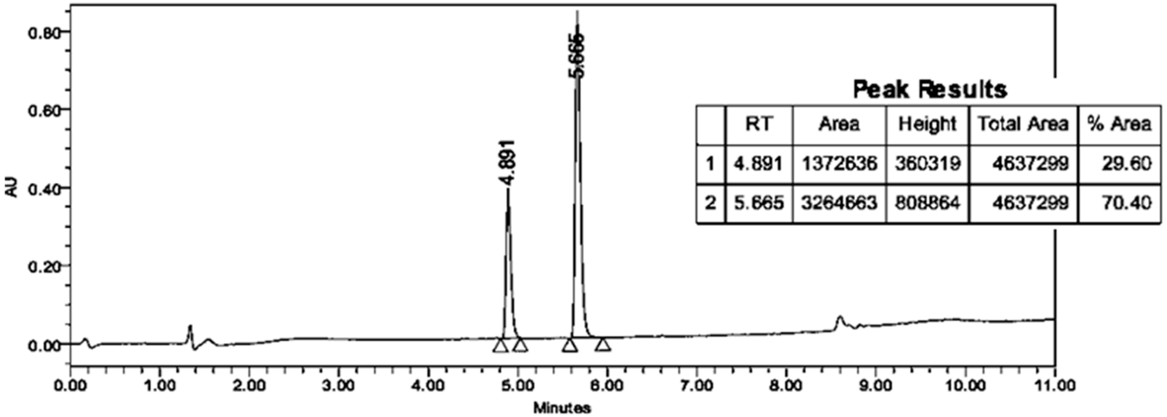

Peak Results

	RT	Area	Height	Total Area	% Area
1	4.891	1372636	360319	4637299	29.60
2	5.665	3264663	808864	4637299	70.40

t_R = 4.89 min → 2,6-dimethoxybenzaldehyde (**15**); t_R = 5.67 min → 2,4-dimethoxybenzaldehyde (**16**)

UV-Vis:

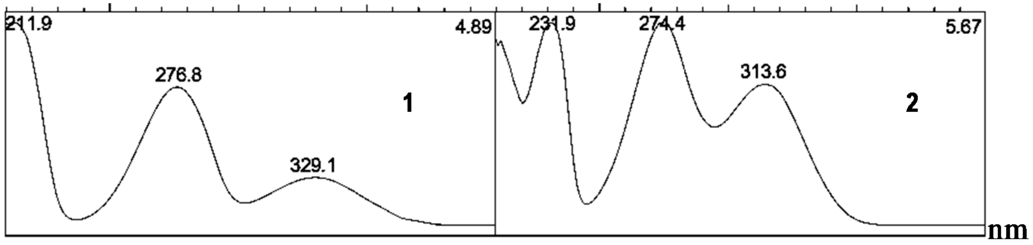

- **Entry 9: Reaction with 1,3,5-trimethoxybenzene:**

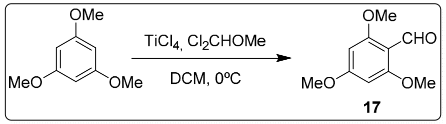

HPLC crude:

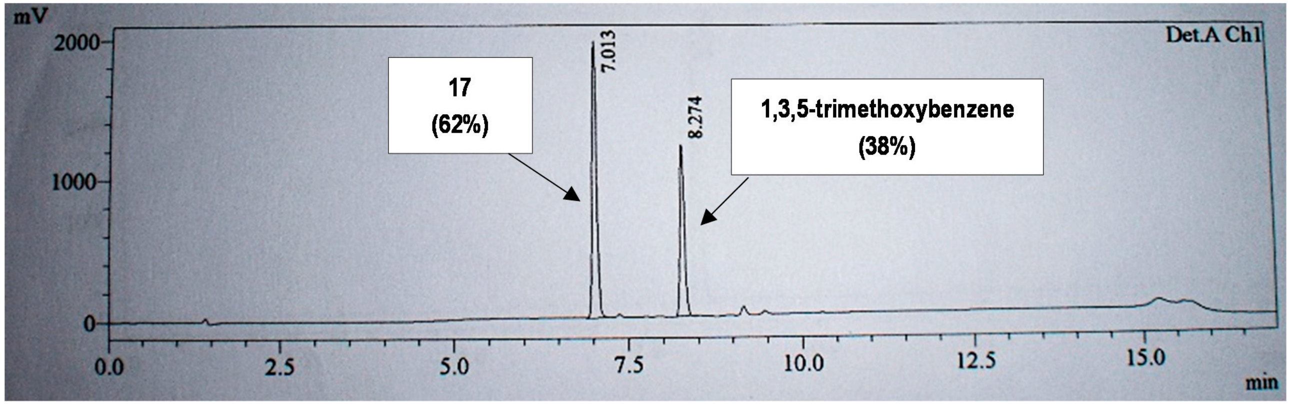

HPLC B* (X-Bridge, C_{18}, 5-100% ACN, t = 11 min

- **Entry 10: Reaction with 3,5-dimethylanisole:**

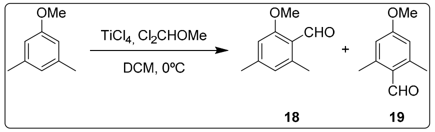

HPLC Crude:

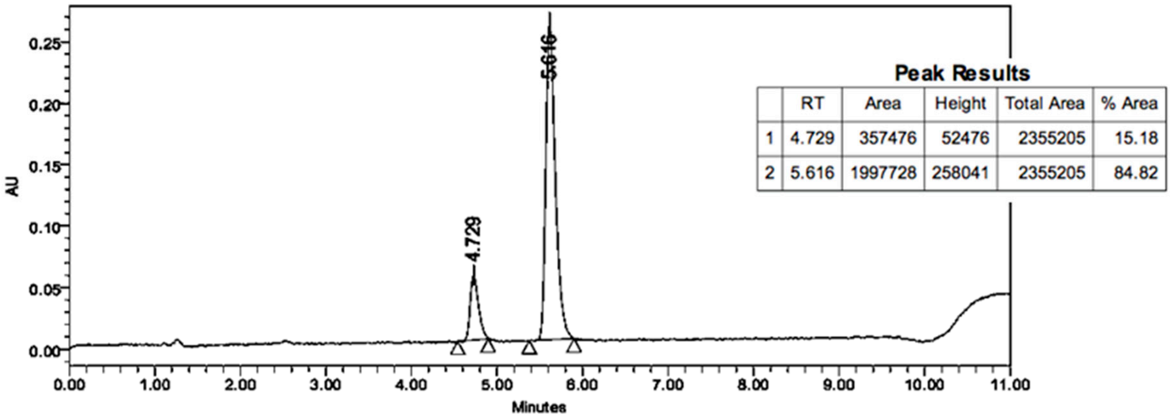

Peak Results

	RT	Area	Height	Total Area	% Area
1	4.729	357476	52476	2355205	15.18
2	5.616	1997728	258041	2355205	84.82

t_R = 4.73 min → 4-methoxy-2,6-dimethylbenzaldehyde (**19**); t_R = 5.62 min → 2-methoxy-4,6-dimethylbenzaldehyde (**18**).

UV-Vis:

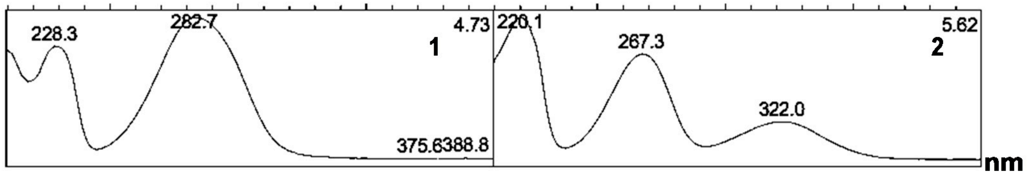

- **Entry 11: Reaction with *o*-xylene:**

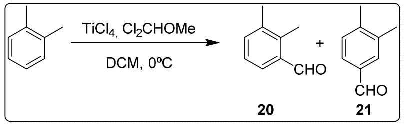

HPLC crude:

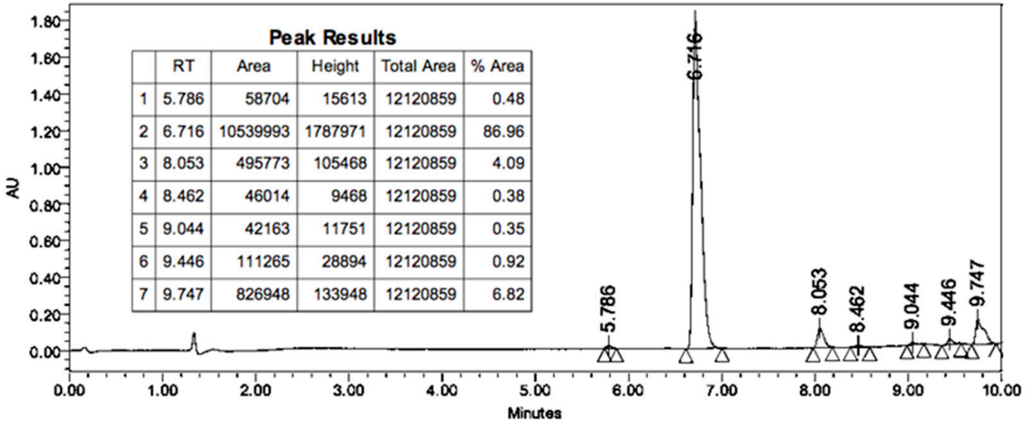

	RT	Area	Height	Total Area	% Area
1	5.786	58704	15613	12120859	0.48
2	6.716	10539993	1787971	12120859	86.96
3	8.053	495773	105468	12120859	4.09
4	8.462	46014	9468	12120859	0.38
5	9.044	42163	11751	12120859	0.35
6	9.446	111265	28894	12120859	0.92
7	9.747	826948	133948	12120859	6.82

t_R = 6.72 min → Regioisomers mixture (**20** and **21**); t_R = 8.05 min → Dimerzation products (HPLC-MS: observed = 223.26 *m/z* correspond to $[M\text{-}OH]^+$)

UV-Vis:

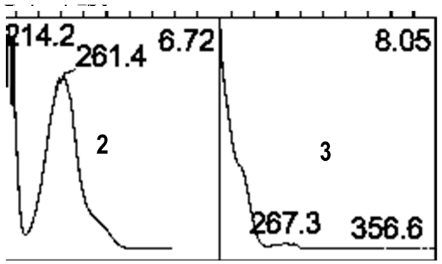

nm

- **Entry 12: Reaction with *m*-xylene:**

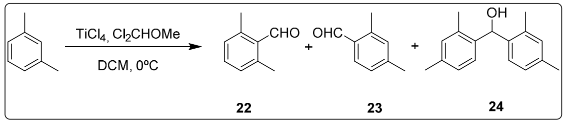

HPLC Crude:

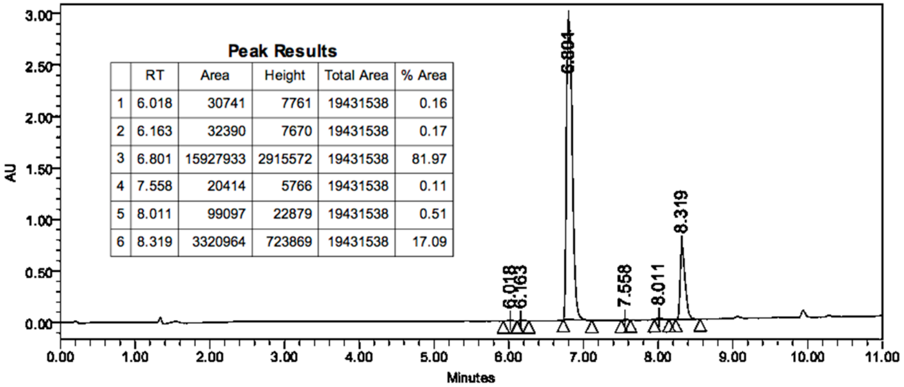

	RT	Area	Height	Total Area	% Area
1	6.018	30741	7761	19431538	0.16
2	6.163	32390	7670	19431538	0.17
3	6.801	15927933	2915572	19431538	81.97
4	7.558	20414	5766	19431538	0.11
5	8.011	99097	22879	19431538	0.51
6	8.319	3320964	723869	19431538	17.09

t_R = 6.801 min → Regioisomeric mixture (**22** and **23**), t_R = 8.319 min → *bis(2,4-dimethylphenyl)methanol* (**24**) (HPLC-MS: observed 223.26 *m/z* corresponding to $[M\text{-}OH]^+$)

UV-Vis:

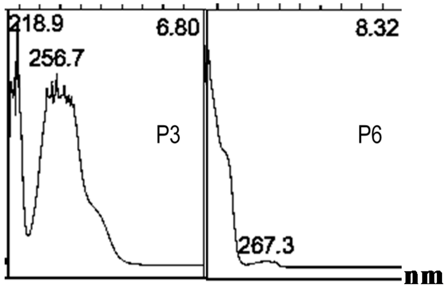

- **Entry 13: Reaction with *p*-xylene:**

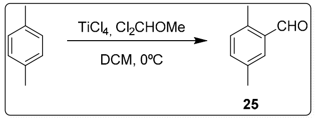

HPLC crude:

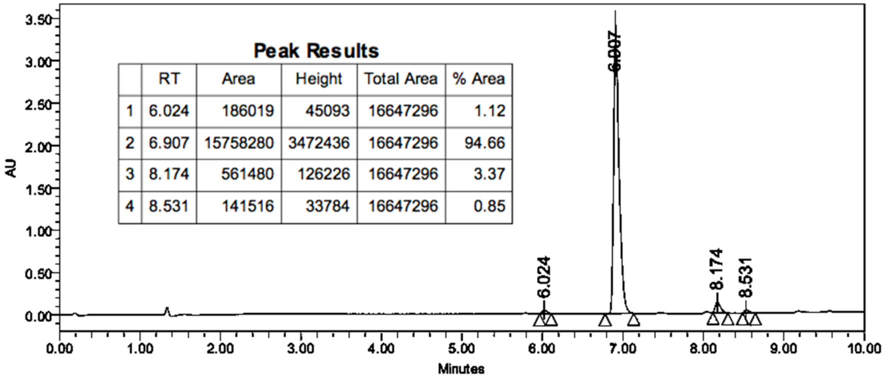

	RT	Area	Height	Total Area	% Area
1	6.024	186019	45093	16647296	1.12
2	6.907	15758280	3472436	16647296	94.66
3	8.174	561480	126226	16647296	3.37
4	8.531	141516	33784	16647296	0.85

t_R = 6.91 min → 2,5-dimethylbenzaldehyde (**25**), t_R = 8.17 min → dimerization product (HPLC-MS: observed 223.26 *m/z* corresponding to $[M\text{-}OH]^+$)

UV-Vis:

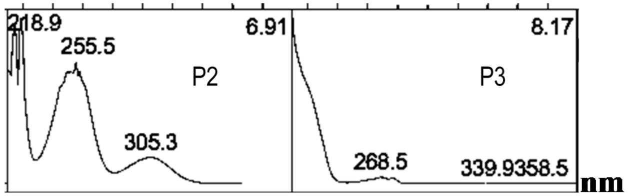

- **Entry 14: Reaction with mesytilene:**

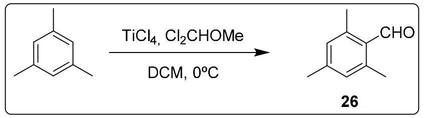

HPLC crude (g30→100 t8 min):

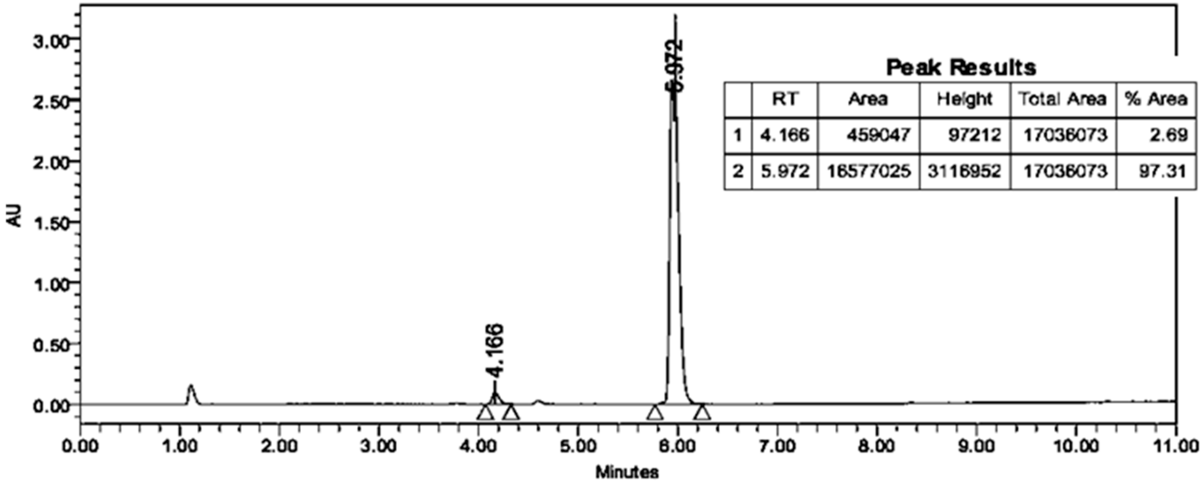

Peak Results

	RT	Area	Height	Total Area	% Area
1	4.166	459047	97212	17036073	2.69
2	5.972	16577025	3116952	17036073	97.31

t_R = 4.17 min → No identified. t_R = 5.97 min → 2,4,6-trimethylbenzaldehyde (**26**)

UV-Vis:

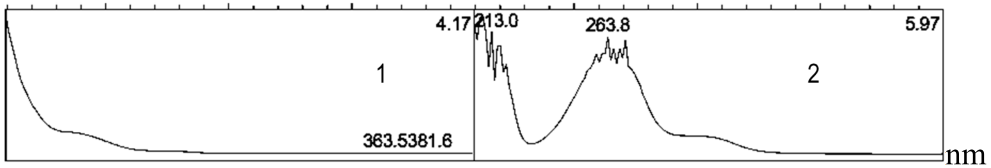

3. Resume tables of formylation reactions.

Table S1. Resume table of phenol formylation.

Entry		Compound		Crude purity (% HPLC)	Product purity (% HPLC)	Yield (%)
**Phenols**						
1	1	2-hydroxybenzaldehyde	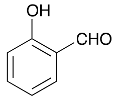	18.0 (t_R = 3.6 min)[1]	—	—
	2	4-hydroxybenzaldehyde	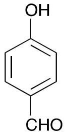	49.9 (t_R = 4.9 min)[1]	>99	0.8 (22)[2]
2	4	2-hydroxy-4-methylbenzaldehyde	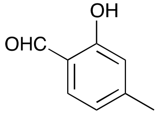	72.3 (t_R = 5.7 min)[1]	98.8	56 (3:1.6:1) [5.6][2]
	3	2-hydroxy-6-methylbenzaldehyde	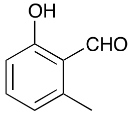			
	5	4-hydroxy-2-methylbenzaldehyde	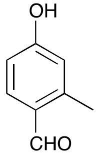	21.2 (t_R = 4.2 min)[1]	>99	

Table S1. *Cont.*

Entry		Compound		Crude purity (% HPLC)	Product purity (% HPLC)	Yield (%)
		**Phenols**				
3	6	6-hydroxy-2-methoxybenzaldehyde	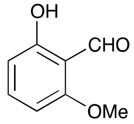	22.7 (t_R = 3.9 min) [1]	19.9	61 (1.3:3.7:1)
	7	2-hydroxy-4- methoxybenzaldehyde	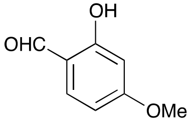	55.7 (t_R = 5.3 min) [1]	77.2	
	8	4-hydroxy-2-methoxybenzaldehyde	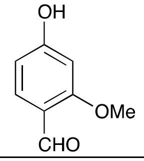	18.7 (t_R = 5.7 min) [1]	96.4	
4	9	2-hydroxy-4,6-dimethylbenzaldehyde	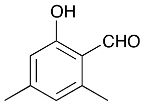	89.9 (t_R = 6.9 min) [1]	>99	78 (5:1)
	10	4-hydroxy-2,6- dimethylbenzaldehyde	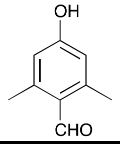	8.2 (t_R = 5.1 min) [1]	>99	

Table S1. *Cont.*

Entry		Compound		Crude purity (% HPLC)	Product purity (% HPLC)	Yield (%)
			**Phenols**			
5	11	6-hydroxy-2,4-dimethoxybenzaldehyde	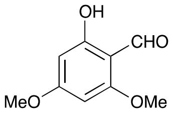	77.8 (t_R = 8.3 min) [4]	99.3	63 (11.1) [2]
		4-hydroxy-2,6- dimethoxybenzaldehyde	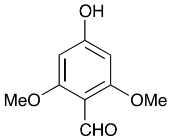	2.6 (t_R = 6.2 min) [4] (t_R = 6.0 min) [3]	—	—
6	12	6-hydroxy-2,3,4-trimethoxybenzaldehyde	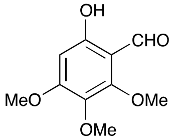	63.5 (t_R = 8.1 min) [4]	96.8	56 (25.3) [2]

[1] HPLC: G05→100 (X-Bridge, C_{18}, 8 min); [2] starting material; [3] HPLC-MS: G05→100 (SunFire C_{18}, 8 min); [4] HPLC: G05→100 (X-Bridge, C_{18}, 11 min).

Table S2. Resume table of methoxybenzene formylation.

Entry	Compound			Crude purity (% HPLC)	Product purity (% HPLC)	Yield (%)
**Methoxybenzenes**						
7	13	2-methoxybenzaldehyde	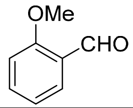	48.5% (t_R = 6.0 min) [1]	—	97 (1:1.1)
	14	4-methoxybenzaldehyde	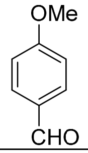	51.5% (t_R = 6.3 min) [1]	—	
8	15	2,6-dimethoxybenzaldehyde	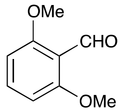	29.6% (t_R = 4.9 min) [1]	>99%	79 (1:3)
	16	2,4-dimethoxybenzaldehyde	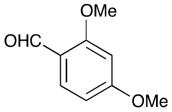	70.4% (t_R = 5.67 min) [1]	>99%	

Table S2. *Cont.*

Entry		Compound		Crude purity (% HPLC)	Product purity (% HPLC)	Yield (%)
**Methoxybenzenes**						
9	17	2,4,6-trimethoxybenzaldehyde	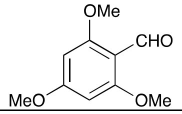	61.8% (t_R = 7.0 min) [4]	95.6%	44 (38.2%) [2]
10	18	2-methoxy-4,6-dimethylbenzaldehyde	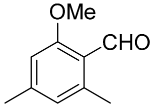	83.8% (t_R = 7.0 min) [1]	>99%	19 (3.5:1)
	19	4-methoxy-2,6-dimethylbenzaldehyde	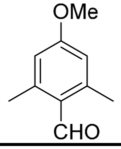	16.2% (t_R = 6.7 min) [1]	>99%	

[1] HPLC: G05→100 (X-Bridge, C_{18}, 8 min); [2] starting material; [4] HPLC: G05→100 (X-Bridge, C_{18}, 11 min).

Table S3. Resume table of methylbenzene formylation.

Entry	Compound			Crude purity (% HPLC)	Product purity (% HPLC)	Yield (%)
**Methylbenzenes**						
11	20	2,3-dimethylbenzaldehyde	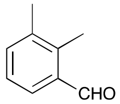	89.2% (t_R =6.7 min) [1]	99.4%	70 (3.2:1)
	21	3,4-dimethylbenzaldehyde	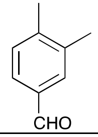			
	-	bis(2,3-dimethylphenyl)methanol	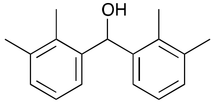	4.4% (t_R =8.1 min) [1]	—	—
	-	bis(3,4-dimethylphenyl)methanol	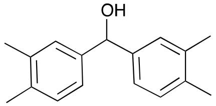		—	—
12	22	2,6-dimethylbenzaldehyde	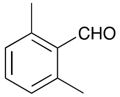	82.0% (t_R =6.8 min) [1]	98.9%	62 (1:32) [0.5] [2]
	23	2,4-dimethylbenzaldehyde	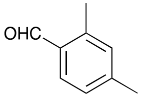			
	24	bis(2,4-dimethylphenyl)methanol	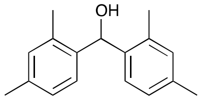	17.1% (t_R =8.3 min) [1]	97.0%	9.9

Table S3. *Cont.*

Entry		Compound		Crude purity (% HPLC)	Product purity (% HPLC)	Yield (%)
**Methylbenzenes**						
13	25	2,5-dimethylbenzaldehyde	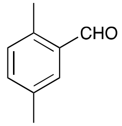	94.7% (t_R = 6.9 min) [1]	—	97
	-	bis(2,5-dimethylphenyl)methanol	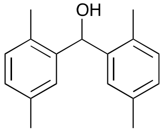	3.4% (t_R = 8.2 min)[1] (t_R = 10.5 min) [3]	—	
14	26	2,4,6-trimethylbenzaldehyde	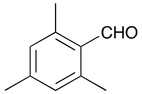	96.7% (t_R = 5.97 min) [5]	—	96

[1] HPLC: G05→100 (X-Bridge, C_{18}, 8 min); [2] starting material; [3] HPLC-MS: G05→100 (SunFire C_{18}, 8 min); [5] HPLC: G30→100 (X-Bridge, C_{18}, 8 min).
